# Effectiveness of biomaterial-based combination strategies for spinal cord repair – a systematic review and meta-analysis of preclinical literature

**DOI:** 10.1038/s41393-022-00811-z

**Published:** 2022-05-23

**Authors:** Alba Guijarro-Belmar, Anna Varone, Martin Rugema Baltzer, Saurav Kataria, Ezgi Tanriver-Ayder, Ralf Watzlawick, Emily Sena, Catriona J. Cunningham, Ann M. Rajnicek, Malcolm Macleod, Wenlong Huang, Gillian L. Currie, Sarah K. McCann

**Affiliations:** 1 School of Medicine, Medical Sciences and Nutrition, Institute of Medical Sciences, University of Aberdeen, Aberdeen, UK; 2 Sainsbury Wellcome Centre, University College London, London, W1T 4JG UK; 3 Centre for Clinical Brain Sciences, University of Edinburgh, Edinburgh, UK; 4 Department of Neurosurgery, Freiburg University Medical Center, Freiburg, Germany; 5 Berlin Institute of Health at Charité—Universitätsmedizin Berlin, QUEST Center, Charitéplatz 1, 10117 Berlin, Germany

**Keywords:** Spinal cord injury, Neurological models, Neurophysiology

## Abstract

**Study design:**

Systematic review and meta-analysis of preclinical literature.

**Objectives:**

To assess the effects of biomaterial-based combination (BMC) strategies for the treatment of Spinal Cord Injury (SCI), the effects of individual biomaterials in the context of BMC strategies, and the factors influencing their efficacy. To assess the effects of different preclinical testing paradigms in BMC strategies.

**Methods:**

We performed a systematic literature search of Embase, Web of Science and PubMed. All controlled preclinical studies describing an in vivo or in vitro model of SCI that tested a biomaterial in combination with at least one other regenerative strategy (cells, drugs, or both) were included. Two review authors conducted the study selection independently, extracted study characteristics independently and assessed study quality using a modified CAMARADES checklist. Effect size measures were combined using random-effects models and heterogeneity was explored using meta-regression with tau^2^, I^2^ and R^2^ statistics. We tested for small-study effects using funnel plot–based methods.

**Results:**

134 publications were included, testing over 100 different BMC strategies. Overall, treatment with BMC therapies improved locomotor recovery by 25.3% (95% CI, 20.3–30.3; *n* = 102) and in vivo axonal regeneration by 1.6 SD (95% CI 1.2–2 SD; *n* = 117) in comparison with injury only controls.

**Conclusion:**

BMC strategies improve locomotor outcomes after experimental SCI. Our comprehensive study highlights gaps in current knowledge and provides a foundation for the design of future experiments.

## Introduction

The inability of adult mammalian Central Nervous System (CNS) neurons to regrow in response to spinal cord injury (SCI) is due to their limited intrinsic regrowth capacity and a hostile post-injury environment [1]. The majority of preclinical SCI repair approaches have been monotherapies, including different pharmacological interventions such as neurotrophic and angiogenic factors, cell therapies, and rehabilitative training [2].

Neurotrophic factors are a heterogeneous group of molecules involved in the development of the CNS and they promote robust neuronal survival and neurite outgrowth in the developing and adult CNS [3]. Early phase clinical trials have tested the efficacy of neurotrophins using gene therapy in patients with neurodegenerative diseases and SCI [4, 5]. One limitation of neurotrophins is that they selectively stimulate the outgrowth of subpopulations of neurons; for example, brain-derived neurotrophic factor (BDNF) promotes axonal regrowth of sensory but not corticospinal neurons [3]. Therefore, multiple trophic factors should be combined for a spinal cord repair therapy and their types and doses should be chosen and optimised carefully [3]. Recently, angiogenesis has been appreciated as a key component of any CNS regenerative strategy because without new blood vessel formation waste products cannot be removed from the injury site and nutrients cannot be provided. Consequently, angiogenic factors such as vascular endothelial growth factor (VEGF) have been used to promote vascularization after SCI [6]. Furthermore, cell therapy is an attractive therapeutic approach for SCI as it can provide significant neuroprotection, recovery through cell replacement, trophic support, and immune modulation [7]. Despite these advantages there are still several challenges such as choice of cell type, cell harvesting and cell differentiation that impede translation of this therapy to the clinic [8]. Studies have suggested that neural stem cells (NSCs) and mesenchymal stem cells (MSCs) exert a clear therapeutic benefit. NSCs can differentiate into neurons or glial cells but autologous NSC transplantation is not readily feasible [9]. MSCs are a more appealing choice because of the ease for autologous transplantation and efficient expansion, yet their utility is confined to immunomodulatory and trophic effects and their neuronal differentiation is questioned [8]. Hence, fundamental questions regarding cell treatments still need to be answered.

**Fig. 1:**
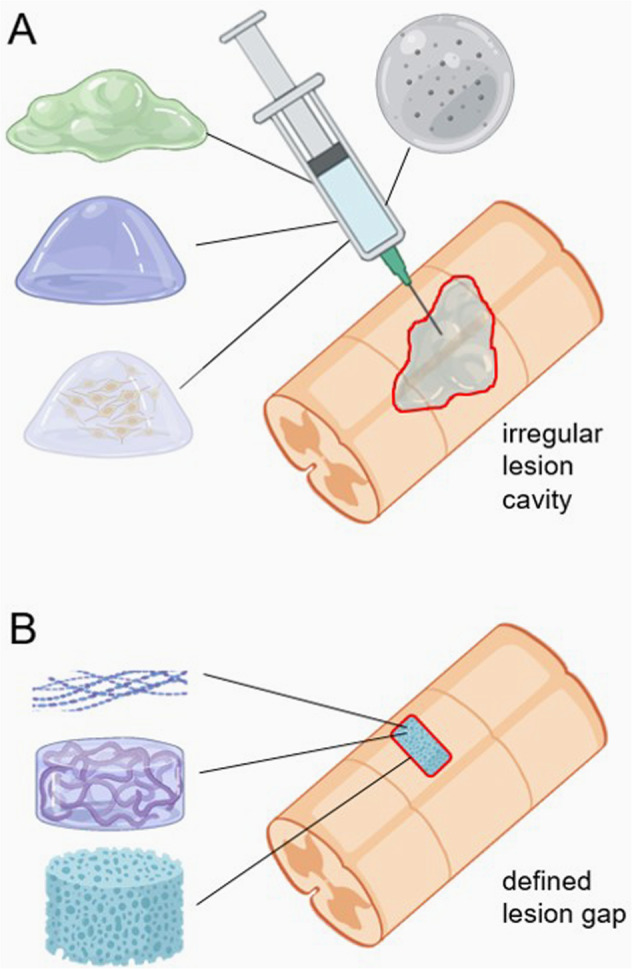
Formats for biomaterials. **A** A spinal cord injury with a large, irregularly shaped lesion site or cavity typical of a crush injury. This injury type is suited to injection of materials, including (counter clockwise, from upper right) a hydrogel loaded with microparticles, an amorphous hydrogel, a soft hydrogel, or a gel seeded with a defined cell type. **B** A smaller, well defined injury site, more typical of a transection injury. This is suited to direct surgical insertion of scaffold materials, including (from top) fibrous materials with aligned or non-aligned matrices, a relatively firm hydrogel with or without a fibrous matrix, or a matrix with a porous character. The cavities in the material may form contiguous channels or be discontinuous. Created with icons from BioRender.com.

However, given the pathophysiological complexity of SCI, any single intervention is unlikely to improve patient outcomes [10]. Instead, combination therapies seem necessary and among these, many are biomaterial-based [11]. Historically, biomaterials for SCI repair have been used because of their ability to provide structural or active growth support to damaged axons. Moreover, biomaterials can act as a delivery platform for cells and therapeutic molecules, and a localised depot for sustained drug release [11, 12]. Ideally, biomaterials for SCI repair should support axonal growth with appropriate stiffness, biocompatibility, and degradability [13, 14]. Moreover, they should be modifiable according to the injury e.g., injectable hydrogels for irregular cavities seen with contusion SCI or implantable scaffolds for defined injuries such as those following laceration SCI (Fig. 1) [13, 14]. They can be natural, synthetic or a mixture of both. Natural biomaterials are widely available and obtained from sources such as plants, animals and DNA. They contain very regular structures due to highly-controlled synthesis and normally exhibit better biocompatibility than synthetic biomaterials. However, owing to their natural origin, they often contain contaminating molecules [15]. Synthetic biomaterials can be easily modified to optimise their mechanical properties and to contain functional sequences for cell signalling. They are also more easily sterilised than natural materials, and their degradation pattern can be controlled [11, 16–18].

Narrative reviews have focused on preclinical research on biomaterials for SCI repair and we have conducted systematic reviews of single therapeutic strategies for traumatic SCI repair [11, 14, 19–21]. However, no systematic and quantitative summary of biomaterial-based preclinical research exists. Therefore, we conducted a systematic review and meta-analysis to assess the evidence for biomaterial-based combination strategies for SCI. Our pre-specified objectives were to assess: (1) the characteristics and effects of the biomaterial only (BMO), when tested in the context of combination strategies for SCI in vitro and in vivo; (2) effects of biomaterial-based combination (BMC) strategies for SCI tested in vitro and/or in vivo and the impact of study quality, study design and publication bias; and (3) whether biomaterial properties and prior in vitro testing have an impact on the effectiveness of BMC strategies in vivo.

## Methods

The study protocol was pre-registered on the CAMARADES website [22] and protocol deviations are described in the supplementary materials. We searched PubMed, Embase and Web of Science on April 28th 2016, and again on May 1st 2018, before data analysis commenced. Titles and abstracts identified in the search were screened independently by two reviewers and discrepancies resolved through discussion. We included all controlled preclinical studies, either in vitro or in vivo, that provided quantitative outcomes and described a BMC strategy that included a non-biomaterial therapy such as cells or drugs. BMO outcomes were also included when they formed part of a study assessing a BMC strategy. For in vivo outcomes, the control was defined as SCI without any treatment. For in vitro outcomes, the control was defined as cell culture, with no treatment added.

Two independent reviewers extracted data, including graphical data, from the included studies, resolving any discrepancies (including ≥ 10% difference in extracted values) via discussion. We extracted study-specific characteristics including biomaterial type/name/structure; animal sex/weight/species; injury type/level; combination strategy, and type of experiment e.g., “in vivo only” or “in vivo, in vitro and biomaterial property”. The primary outcomes were in vivo locomotor recovery and in vitro and in vivo axonal regeneration (not including axonal sprouting). Inclusion/exclusion criteria and primary and secondary outcomes are further described in the supplementary material.

We extracted group-level data for SCI with treatment, SCI without treatment (control), and uninjured (sham) groups. For each outcome we extracted the number of animals or samples, outcome mean, and the Standard Error of the Mean (SEM) or Standard Deviation (SD) in each group, the time of intervention and the assessment time. We extracted outcomes from individual components of the combination if reported, specifying each comparison as “effect of combination”, “effect of biomaterial”, “effect of drug”, or “effect of cells”. Full names of abbreviated biomaterials, drugs and cells are described in the supplementary material.

We assessed study quality using a modified CAMARADES checklist [23] comprising evaluation of: randomisation, allocation concealment, blinding, sample size calculation, animal welfare compliance, potential conflicts of interest, and animal exclusions (e.g., deaths, surgical failure). For each comparison between a treatment and a control group, we calculated an effect size. For in vivo locomotor comparisons we calculated a normalised mean difference (NMD) [21, 24], presented as percentage improvement in the treatment vs. control group. For all other comparisons, we calculated a standardised mean difference (SMD), presented as improvement in outcome in the treatment *vs*. control group, in SD units. We pre-specified a minimum of 25 independent comparisons needed to perform meta-analysis on the primary and secondary outcomes. We combined effect size measures using random-effects models with restricted maximum likelihood (REML) estimate of between-study variance. the combination of heterogeneous studies In preclinical systematic reviews, means that often the meta-analytic pooled estimate of effect is less important than examining the sources of heterogeneity: identifying the factors contributing to between-study differences and what they can tell us about the efficacy of the intervention under different conditions. To assess heterogeneity, we used tau^2^ (between-study variance), I^2^ (percentage of variation attributable to between-study heterogeneity) and adjusted R^2^ (adjR^2^; proportion of between-study variance explained by the covariate). Using univariate meta-regression, we evaluated the impact of the study design variables we pre-defined in our protocol. These included the variables related to risks of bias and internal validity that we assessed using the modified CAMARADES checklist (referred to as study quality variables, listed above), in addition to the following study design variables: animal type and sex, type and level of injury, time of assessment and administration of analgesia. Where the number of comparisons was sufficient (10 independent comparisons per variable included in the model), we also used multivariable meta-regression. Each study design or study quality variable contained two or more levels (e.g., true, false, not reported). Where one level of a binary variable contained >90% of comparisons, we did not carry out meta-regression. Where comparisons were unbalanced in a variable with more than two levels, we grouped levels with <5 comparisons into an “Other” level. For combination strategies, variable levels were grouped based on the biomaterial used, e.g., studies using collagen-based biomaterials combined with other strategies were grouped into the “collagen + combination” level. Meta-regression was conducted on datasets with grouped comparisons.

Holm-Bonferroni adjusted critical *p* values were used to adjust for the number of univariate meta-regression analyses per objective and dataset. We assessed the presence of small-study effects using funnel-plots, Egger’s regression, and trim-and-fill. Small- study effects describes the phenomenon where smaller studies are often associated with larger treatment effects, potentially due to publication bias. All statistical analyses were performed using Stata (Release 16; StataCorp LP, USA).

## Results

We identified 2068 publications in the literature search (eFig. 1), of which 134 were included (eTable 1).

### Objective 1: characteristics and effects of biomaterials used in combination strategies

**Fig. 2:**
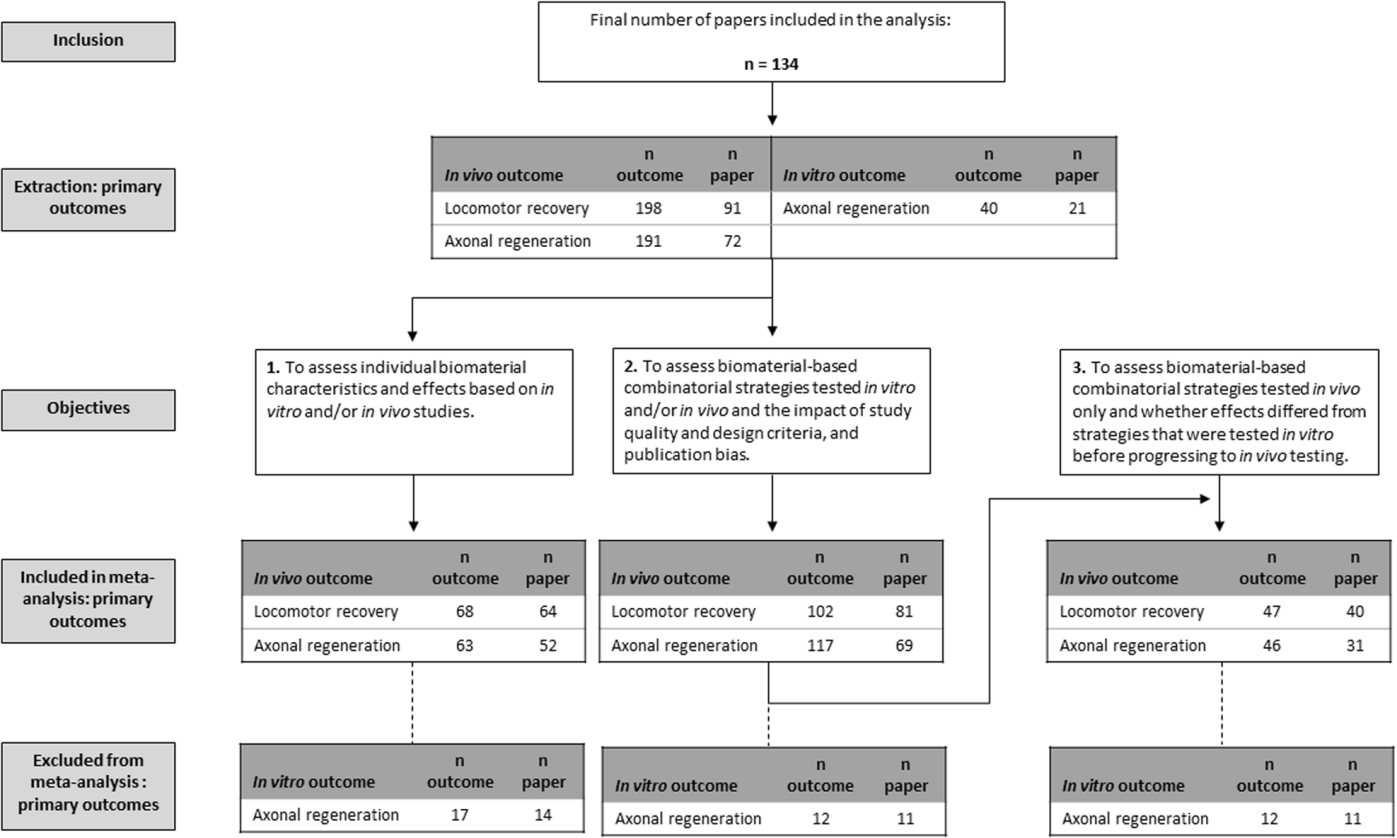
Flow diagram of included studies. Data from 134 publications were included in the meta-analysis and study quality/design assessment. Following data extraction, the analysis was conducted based on the set objectives. Of the included studies, 91 papers reported locomotor recovery outcomes, 72 reported in vivo axonal regeneration outcomes and 21 reported in vitro axonal regeneration. Objective 1 includes only comparisons that assessed the effect of biomaterials alone. Objective 2 includes studies that assessed BMC strategies in vitro, in vivo, and/or studied the biomaterial properties. Objective 3 includes studies that carried out investigations only in vivo.

We first analysed biomaterial-specific outcomes, where BMO effects in SCI models were established independently of combination strategies. We identified 68 and 63 comparisons for locomotor recovery and in vivo axonal regeneration, respectively (Fig. 2). As only 17 comparisons were identified for in vitro axonal regeneration, no further analysis was conducted. eTable 2 summarises 58 comparisons for secondary outcomes. BMO treatment improved locomotor recovery by 7.9% (95% confidence interval [CI] 4.9–11, *p* < 0.0001, Tau^2^ = 83.6, I^2^ = 90.4%, *n* = 68) and in vivo axonal regeneration by 1.1 SD (95% CI 0.7–1.5, *p* < 0.0001, Tau^2^ = 1.4, I^2^ = 77.3%, *n* = 63). Significant heterogeneity was found but could not be explained by biomaterial type (locomotor recovery: *p* = 0.691, Tau^2^ = 85.3, I^2^ = 89.7%, adjR^2^ = 0%; eFigure 2A and in vivo axonal regeneration: *p* = 0.959, Tau^2^ = 1.5, I^2^ = 78.3%, adjR^2^ = 0%). For locomotor recovery outcomes, 57%, 24% and 19% of biomaterials were identified as natural, synthetic, or mixed, respectively (eFig. 2A). Biomaterial format had no effect on locomotor recovery (*p* = 0.610, Tau^2^ = 89.4, I^2^ = 88.4%, adjR^2^ = 0%, Table 1A). Scaffold was the most commonly used format (33.8% of comparisons) and conferred a 10.4% improvement (95% CI 5.2–15.6%; Table 1A) in locomotor recovery. This was followed by non-injected hydrogel (used in 27.9% of comparisons). Thirty-two individual biomaterials were assessed for their effects on locomotor recovery and 30 for in vivo axonal regeneration. Thirty-seven percent of locomotor recovery and 59% of in vivo axonal regeneration comparisons evaluated individual biomaterials that were tested in fewer than 5 experiments (grouped as “Other”; Table 1B, C). No significant relationships existed between the biomaterial used and locomotor recovery (*p* = 0.510, Tau^2^ = 78.4, I^2^ = 87.2%, adjR^2^ = 6.3%; Table 1B) or in vivo axonal regeneration (*p* = 0.245, Tau^2^ = 1.4, I^2^ = 76.7%, adjR^2^ = 0.41%; Table 1C). Multivariable meta-regression including biomaterial type and format was conducted but could not explain a significant proportion of the heterogeneity in locomotor recovery (*p* = 0.814, Tau^2^ = 89.4, I^2^ = 85.4%, adjR^2^ = 0%) or in vivo axonal regeneration (*p* = 0.256, Tau^2^ = 1.4, I^2^ = 76.5%, adjR^2^ = 0%; eTable 3). Most analyses contained insufficient data to draw definitive conclusions about efficacy of and differences between biomaterials, regardless of type and format. eFigure 2B–D provides the effect sizes of all biomaterials, illustrating high within-group variability.

**Table 1:** Objective 1, meta-regression analysis of the effect of (A) the biomaterial format, (B) the specific biomaterial on locomotor recovery and (C) the specific biomaterial on in vivo axonal regeneration in BMO studies.

A Improvement in locomotor outcomes				
Biomaterial format	Effect size (%)	*P* > | t |	95% Conf. Interval	Frequency % (n)
Scaffold	10.4	0.001	[5.2, 15.6]	33.8 (23)
Microsphere-loaded hydrogel	9.6	0.967	[−3.8, 22.9]	8.8 (6)
Hydrogel (not injected)	8.9	0.695	[1.1, 16.7]	27.9 (19)
Linear oriented scaffold	4.6	0.189	[−4.2, 13.4]	19.1 (13)
Hydrogel (injected)	1.4	0.120	[−10.2, 13]	8.8 (6)
Other formats	8.7	0.907	[−20, 37.5]	1.5 (1)
	comparisons= 68, *p* = 0.610, Tau^2^ = 88.43, I^2^ = 88.43%, adj R^2^ = 0%			

B Improvement in locomotor outcomes				
Biomaterial name	Effect size (%)	*P* > | t |	95% Conf. Interval	Frequency % (n)
PHEMA-MMA	12	0.553	[−2.2, 26.2]	7.3 (5)
PLGA	8.7	0.875	[−3.8, 21.3]	8.7 (6)
Collagen	7.8	0.054	[−0.2, 15.7]	20.6 (14)
HA	6.6	0.863	[−7.1, 20.3]	7.4 (5)
Chitosan	4.7	0.578	[−6.3, 15.6]	11.8 (8)
HAMC-PLGA	−0.8	0.196	[−13.9, 12.3]	7.4 (5)
Other biomaterials	10.7	0.001	[1.3, 20]	36.8 (25)
	comparisons= 68, *p* = 0.510, Tau^2^ = 78.4, I^2^ = 87.2%, adj R^2^ = 6.28%			

C Improvement in axonal regeneration				
Biomaterial name	Effect size (SD)	*P* > | t |	95% Conf. Interval	Frequency % (n)
PLGA	0.9	0.901	[−0.6, 2.4]	9.5 (6)
Collagen	0.8	0.076	[−0.1, 1.6]	22 (14)
HA-PLGA	0.1	0.412	[−1.4, 1.7]	9.5 (6)
Other biomaterials	1.4	0.207	[0.4, 2.5]	59 (37)
	comparisons= 63, *p* = 0.240, Tau^2^ = 1.4, I^2^ = 72%, adj R^2^ = 0.41%			

*PHEMA-MMA* poly(2-hydroxyethyl methacrylate-comethylmethacrylate), *PLGA* poly(lactic-co-glycolic-acid), *HA* hyaluronic acid, *HAMC* hyaluronic acid methylcellulose.

### Objective 2: biomaterial-based combination strategies tested in vitro and/or in vivo

The analyses for this objective included all data from studies testing BMC strategies, i.e. in vitro evaluation before in vivo testing and in vivo testing only. We identified 102 and 117 comparisons for locomotor recovery and in vivo axonal regeneration, respectively (Fig. 2). As only 12 comparisons were identified for in vitro axonal regeneration, no further analysis was conducted. eTable 4 summarises 63 secondary outcomes. BMC treatments significantly enhanced locomotor recovery by 25.3% (95% CI 20.3–30.3%, *p* < 0.0001, Tau^2^ = 543, I^2^ = 98.4%, *n* = 102), and in vivo axonal regeneration by 1.6 SD (95% CI 1.2–2 SD, *p* < 0.0001, Tau^2^ = 2.5, I^2^ = 86.3%, *n* = 117). Treatment effects of different BMC strategies on behavioural and histological outcomes are detailed in eFigures 3A–C, 4A–C. Seventy-two combinations were assessed for their effect on locomotor recovery and 64 for in vivo axonal regeneration (eFigs. 2–3). Outcomes were grouped according to biomaterial for meta-regression (Table 2) but we did not find significant effects of combinations (locomotor recovery: *p* = 0.142, Tau^2^ = 520.7, I^2^ = 97.7%, *n* = 102; in vivo axonal regeneration: *p* = 0.124, Tau^2^ = 2.2, I^2^ = 84.1%, *n* = 117). Poly(lactic-co-glycolic acid)(PLGA)-based and chitosan-based combinations had large effects on in vivo axonal regeneration [2.8 SD (95% CI 0.7–4.8 SD) and 2.9 SD (95% CI 0.9–4.8 SD); Table 2B] compared to control. PGLA-based combinations also had a large effect on locomotor recovery [41.5% (95% CI 16.7–66.3%); Table 2A]. Most other combinations, grouped according to biomaterial, had no measurable effects on in vivo axonal regeneration (Table 2B).

**Table 2:** Objective 2, meta-regression analysis of the effect of BMC strategies on (A) locomotor recovery and (B) in vivo axonal regeneration; combinations tested in vitro and/or in vivo.

A Improvement in locomotor outcomes				
Biomaterial-based combination	Effect size (%)	*P* > | t |	95% Conf. Interval	Frequency % (n)
PLGA + combinations	41.5	0.064	[16.7, 66.3]	4.9 (5)
Chitosan + combinations	27.3	0.289	[10.2, 44.4]	13.7 (14)
HA + combinations	22.5	0.684	[1.1, 43.9]	6.9 (7)
Collagen + combinations	18.1	0.002	[6.9, 29.2]	22.6 (23)
Fibrin + combinations	14.8	0.703	[−2.1, 31.7]	13.6 (14)
Other biom. + combinations	30.7	0.064	[17, 44.4]	37.9 (39)
	comparisons=102, *p* = 0.142, Tau2 = 520.7, I2 = 97.7%, adj R2 = 4.11%			

B Improvement in axonal regeneration				
Biomaterial-based combination	Effect size (SD)	*P* > | *t* |	95% Conf. Interval	Frequency % (n)
Chitosan + combinations	2.9	0.235	[0.9, 4.8]	6 (7)
PLGA + combinations	2.8	0.289	[0.7, 4.8]	5.1 (6)
LOCS + combinations	2.4	0.391	[0.7, 4.0]	6.8 (8)
Alginate + combinations	1.9	0.838	[−0.4, 4.1]	4.27 (5)
Collagen + combinations	1.7	0.001	[0.8, 2.5]	24 (28)
Matrigel + combinations	1.4	0.836	[−0.6, 3.5]	4.3 (5)
Fibrin + combinations	0.3	0.067	[−1.2, 1.8]	8.5 (10)
Fibrin-PLGA + combinations	−0.1	0.078	[−2, 1.8]	5.1 (6)
Other biom. + combinations	1.6	0.853	[0.5, 2.7]	35.9 (42)
	comparisons = 117, *p* = 0.182, Tau^2^ = 2.2, I^2^ = 76.2%, adj R^2^ = 9.3%			

*PLGA* poly(lactic-co-glycolic-acid), *HA* hyaluronic acid, *LOCS* linear ordered collagen scaffold.

**Fig. 3:**
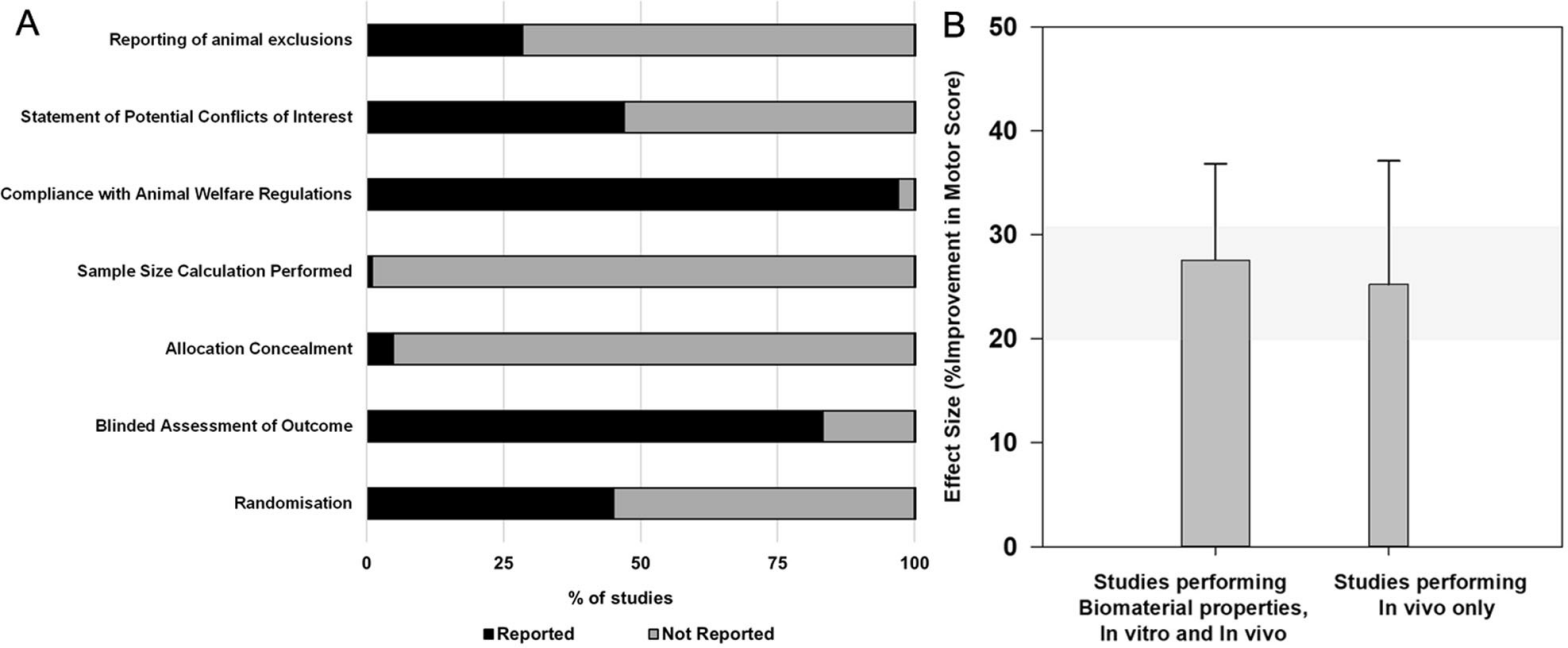
Influence of the testing paradigm used on locomotor recovery outcomes and percentage of reporting study quality parameters. **A** Percentage of studies reporting study quality parameters. **B** Effect of the influence of testing biomaterial properties and performing in vitro and in vivo experiments testing combinations (*n* = 29) vs. in vivo experiments only (*n* = 47) on the effect size as a percentage of improvement in motor score. Vertical error bars represent the 95% CI for the individual estimates, and the horizontal shaded grey bar represents the 95% CI of the global estimate. The width of each vertical bar is normalised to the square root of number of animals contributing to that comparison.

We next investigated the effect of seven study quality items on locomotor recovery outcomes (Fig. 3A; eFig. 5). Blinding and randomisation were reported in 83.3% and 45.1% of comparisons, respectively. Few studies provided a description of the randomisation method (*n* = 20/86). Only 47.1% and 28.4% of comparisons provided conflict of interest statements and animal exclusions, respectively. Allocation concealment and sample size calculation were rarely reported (4.9% and 1.0%, respectively; Fig. 3A). The average animal numbers per group was 10.6 ± 12.1 for control and 10.9 ± 12.4 for treatment. No quality measure was significantly associated with locomotor recovery (eFig. 5A–D).

SCI level of injury was a significant source of heterogeneity (*p* = 0.008, Tau^2^ = 488.1, I^2^ = 97.8%, *n* = 102; eFig. 6D). Mid-thoracic level SCI injury was most commonly used and accounted for 85% of comparisons. In these models treatment improved locomotor recovery by 26.8% (95% CI 21.7–32). Eleven percent of comparisons involved cervical level injury, however in these models treatment had no significant effect on locomotor recovery (6.4% improvement; 95% CI –8.9 to 21.7). The variables sex, post-surgical analgesia, and SCI type (contusion, compression, transection and hemisection) were not significant sources of heterogeneity (eFig. 6A–D). The most common last assessment time points were 8 (24%) and 4 weeks (18%) post-SCI. Transection and hemisection SCIs had the highest frequency (44% and 38%, respectively). For species and sex, rodents and females as animal models accounted for 95% and 70% of comparisons, respectively; no differences were found in locomotor recovery between sexes.

The majority of included studies (98%) administered the BMC treatment acutely, straight after injury or briefly after it (0–7 days after injury), with only 2% of studies applying the treatment in a subacute manner (14 or more days after injury).

No evidence of small-study effects was found using Egger’s regression test. Furthermore, with trim-and-fill analysis we did not detect any theoretically missing studies.

### Objective 3: effects of biomaterial-based combinations tested in vivo only vs. a full testing paradigm

Finally, we sought to determine whether prior in vitro assessments of biomaterial characteristics were associated with a greater improvement in in vivo outcomes after BMC treatment. “In vivo testing only” refers to studies where the authors did not present or reference previous in vitro work characterising biomaterials as part of the rationale for their in vivo experiments testing BMC strategies. The effects of BMC strategies that underwent “in vivo testing only” were assessed in 47/103 in vivo locomotor recovery outcomes and 46/117 in vivo axonal regeneration outcomes from Objective 2 (Fig. 2). Overall, BMC strategies tested only in vivo significantly improved locomotor recovery by 25.3% (95% CI 18.3–32.3%, *p* < 0.0001, Tau^2^ = 466.3 and I^2^ = 97.8%, *n* = 47) and axonal regeneration by 1.3 SD (0.7–2.1 SD, *p* < 0.0001, Tau^2^ = 2.3, I^2^ = 88.1%, *n* = 46). The specific BMC treatment (grouped according to biomaterial; Table 3) was a significant source of heterogeneity (locomotor recovery: *p* = 0.006, Tau^2^ = 318.4, I^2^ = 95.9%, *n* = 47). PLGA-based combinations showed the greatest improvement in locomotor recovery (46.3%, 95% CI 26.4–66.3%, *p* = 0.002; Table 3A). Treatment effects of different BMC strategies on behavioural and histological outcomes are detailed in (eFigure 7).

**Table 3:** Objective 3, meta-regression analysis of the effect of BMC strategies on (A) locomotor recovery and (B) in vivo axonal regeneration; combinations tested in vivo only.

A Improvement in locomotor outcomes				
Biomaterial-based combination	Effect size (%)	*P* > | *t* |	95% Conf. Interval	Frequency % (n)
PLGA + combinations	46.3	0.002	[26.4, 66.3]	12.8 (6)
Chitosan + combinations	19.8	0.476	[0.8, 38.8]	17 (8)
Collagen + combinations	13	0.055	[−0.3, 26.4]	27.7 (13)
Fibrin + combinations	11.3	0.845	[−67, 29.3]	19.1 (9)
Other biom. + combinations	40.1	0.004	[22.3, 57.8]	23.4 (11)
	comparisons=47, *p* = 0.0006, Tau^2^ = 318.4, I^2^ = 95.9%, adj R^2^ = 91.72%			

B Improvement in axonal regeneration				
Biomaterial-based combination	Effect size (SD)	*P* > | *t* |	95% Conf. interval	Frequency % (n)
Chitosan + combinations	2.1	0.263	[−0.5, 4.8]	10.9 (5)
PLGA + combinations	1.7	0.348	[−0.5, 3.9]	19.6 (9)
Matrigel + combinations	1.5	0.53	[−1, 4]	10.9 (5)
Fibrin + combinations	0.7	0.326	[−0.7, 2]	21.7 (10)
Collagen + combinations	0.5	0.869	[−1.6, 2.6]	19.6 (9)
Other biom. + combinations	2.8	0.069	[0.5, 5.1]	17.4 (8)
	comparisons=46, *p* = 0.398, Tau^2^ = 2.56, I^2^ = 88.5%, adj R^2^ = 0%			

*PLGA* poly(lactic-co-glycolic-acid).

A testing paradigm where BMO properties were assessed and the BMC was tested in vitro, prior to in vivo testing, did not result in a greater improvement in locomotor recovery (27.5%, 95% CI 18.3–36.8%, *n* = 29; *p* = 0.696, Tau^2^ = 526.2, I^2^ = 97.72% and adjR^2^ = 0%) than combinations that were tested in vivo only (25.2%, 95% CI 13.3–37.1, *n* = 47; Fig. 3B).

Lastly, we conducted post-hoc analysis of the influence of treatment type (BMO, individual therapies, or BMC) on locomotor recovery outcomes from all included studies. The type of treatment had a significant effect on locomotor recovery (*p* < 0.0001, Tau^2^ = 351.7, I^2^ = 97.5%, adjR^2^ = 14%; *n* = 198; eFig. 8). Biomaterial-based combination treatments resulted in the greatest improvement in locomotor recovery compared to SCI only control [25.3% (95% CI 21.2–29.5); *n* = 102]. This was followed by drugs alone [19.9% (95% CI 8.6–31.2); *n* = 15], cells alone [12.8% (95% CI -0.1 to 25.8); *n* = 13], and biomaterials alone [8.7% (95% CI 2.1–15.2); *n* = 68] compared to SCI only control.

## Discussion

Several reviews have identified and evaluated potential biomaterial-based therapies for SCI repair [14, 20, 25, 26] but none have described the impact of the biomaterial and biological and experimental design factors on efficacy in a transparent summary of all available data. We have investigated three factors important in identifying promising BMC strategies for SCI: biomaterial properties, effectiveness of the combination strategy, and most effective preclinical testing paradigm.

Treatment with BMO resulted in a significant improvement in locomotor recovery and axonal regeneration. However, the specific biomaterial, biomaterial type and format could not explain the significant heterogeneity observed. This is likely due in part to the high number of different biomaterials used relative to the total number of studies: most individual biomaterials were tested in fewer than five comparisons. Additionally, there were low animal numbers reported per study, resulting in imprecise estimates of effect. Our analysis showed that natural biomaterials were used most frequently, likely explained by their excellent biocompatibility, mechanical and degradation properties, and ability to initiate neovascularisation [26]. Synthetic biomaterials were less commonly used but have exceptional properties, including high water content, mechanical stability, and ease of chemical modification to include integration of cell adhesion peptides [16, 17]. However, they are not easily cleared after degradation, which should be a focus area for future research [18]. It was interesting to note that hydrogels were the most frequently used biomaterial format. This technology offers the advantage of creating a complex and precise 3D geometry that conforms exactly with the lesion cavity [27]. Based on the diverse mechanical and biological features of different biomaterials, these factors are likely important determinants of success in combination strategies, and our results highlight an area where more research is needed to draw definitive conclusions regarding relative efficacy.

The SCI research community has reached a consensus that a combination therapeutic strategy is a necessity for SCI repair [26, 28]. However, this agreement was not supported by quantitative data. Our findings add weight to the consensus, by demonstrating that combination treatments improve locomotor recovery by 25.3% and in vivo axonal regeneration by 1.6 SD. It appears that biomaterial-based combination strategies are more effective than cell- or drug-based single strategies. However, this finding should be interpreted with caution, as we did not review all available evidence for these single strategies and this analysis was post-hoc.

Our findings support the potential of BMC approaches to tackle the physical and chemical barriers to SCI repair as well as the lack of intrinsic capacity of adult CNS neurons to regrow. The results of these BMC studies indicate that a biomaterial can be used not only as a permissive substrate to encourage injured axons to regrow but also as a delivery mechanism for cells and drugs. For example, Teng et al. implanted a polymer scaffold combined with NSC, which promoted functional recovery in an adult rat hemisection SCI model [29]. Overall, combinations based on PLGA resulted in robust improvements in outcomes. This US Food and Drug Administration (FDA)-approved synthetic biomaterial [30] has been studied in a variety of forms, including guidance channels, microsphere-loaded hydrogels and scaffolds [17, 31, 32], because of its excellent biocompatibility and degradability profile. Interestingly, PLGA was the biomaterial used in the only paper included in our review using non-human primates as a preclinical animal model [17]. The specific combination used in this study (PLGA and neural stem cells) was previously used in other studies using rats [29, 33], also included in this systematic review and meta-analysis. Interestingly, the only ongoing clinical trial with BMO for SCI repair uses Neuro-Spinal Scaffold™, which is a PLGA-based biomaterial [34]. However, no clinical trial using a biomaterial-based combination has been reported yet. Limitations to clinical translation would likely include regulatory obstacles such as the requirement by the FDA to show efficacy in human patients of not only the biomaterial alone but also the individual efficacy of any other non-biomaterial BMC components.

We did not find a significant difference in the efficacy of BMC strategies where biomaterial properties and in vitro efficacy were evaluated before in vivo experiments, compared to studies where only in vivo testing was carried out. This deserves further investigation when more researchers adopt the *prior* testing/evaluating approach. We advocate such an approach as it would ensure that unsuitable biomaterials do not move into in vivo testing for SCI repair, reducing research waste and contributing to more 3Rs (Replacement, Reduction and Refinement)-aligned research.

Preclinical SCI models have historically included rodents, cats, dogs and non-human primates. We found that rats were most commonly used in these studies, likely due to their small size, ease of handling, and the many well-developed, robust locomotor tests available to assess recovery [35]. Recently, the use of non-human primates in SCI repair research has received greater attention, especially to validate strategies with promise for clinical translation [36, 37]. However, the ethical concerns and financial challenges of using primates remain serious obstacles. We found that mid thoracic region injury was commonly used, and we suggest future research include more cervical models, as human SCI commonly occurs at the cervical region [38, 39]. We also observed that transection and hemisection were most frequently studied in animals, despite contusion being the most common injury type in humans. We understand that for biomaterial-based combination studies transection is more amenable at the early experimental stages, due to the less complex surgery, better postoperative recovery, and easier control of the cavity size. However, we suggest that subsequent testing also incorporate contusion models.

We found that steps to reduce the risk of bias were not a significant source of heterogeneity in the data. The prevalence of randomisation and blinding in our study is higher than that previously observed [21, 40], providing confidence in the findings reported here. We found few studies reported sample size calculations. This is a concern as too-small sample sizes can lead to imprecision and low reproducibility, while too-large sample sizes result in a waste of resources and excessive animal use [41]. We recommend the use of tools including the UK National Centre for the Replacement, Refinement and Reduction of Animals in Research (NC3Rs) Experimental Design Assistant for preclinical study design [42].

### Limitations

Our research question was broad, encompassing all in vitro and in vivo research on BMC strategies investigated for SCI repair. This approach, while providing a comprehensive overview of the field, has limited more specific conclusions. Importantly, In vitro models can only ever mimic certain aspects of SCI and what we infer from these experiments must be informed by an understanding of their biological and pathophysiological limitations. Moreover, there are currently no experimental validity standards for in vitro models in SCI research. Our review provides a comprehensive overview of models used in the context of biomaterial-related research that can contribute to generating such standards within the community.

In general, we found high variability between studies and a lack of data for many strategies that have not been tested in a sufficient manner. This limited our ability to draw robust conclusions about the relative efficacy of BMC strategies, and very little of the observed heterogeneity in the datasets was explained by the variables investigated. However, it may be that unreported or unmeasurable variables contribute to this heterogeneity e.g., noise level in the animal house or method of handling animals. Due to the high number of different biomaterials and combinations studied, we grouped data for meta-regression based on the biomaterial used. For combinations, we were therefore unable to examine potential differences in strategies using cells, drugs or both. Even after grouping, 38% of comparisons evaluated BMC strategies that were tested in fewer than five locomotor experiments.

A broader limitation of these approaches is their relatively low statistical power when the number of included studies is modest [43]. Several outcomes were not analysed as the minimum number of required comparisons was not reached. A general limitation of systematic review and meta-analysis is that these tools can be used to summarise available evidence but cannot overcome deficiencies in quality, reporting or scope, instead only highlighting where gaps in evidence exist. Further, these approaches cannot correct reporting biases, including selective and incomplete reporting and publication bias [44]. In the studies included in the current review, key experimental features were often not reported, including for what purpose a biomaterial was synthesised or isolated, and what type of barrier(s) to neural repair and/or functional recovery it aimed to overcome. This limited our ability to gain insights into the biological processes targeted by different biomaterials and investigate which type(s) of biomaterials produced more reliable results in the context of different injury models.

## Conclusion

Our study provides a comprehensive summary of biomaterial-based combination strategies tested in preclinical SCI models. We demonstrate the effectiveness of these strategies overall for improving locomotor recovery and axonal regeneration. A diverse range of combination strategies has been tested and, while some appear more promising than others, a lack of evidence for many biomaterials and combinations limits our ability to draw definitive conclusions about their relative efficacy. Importantly, we highlight where gaps exist in our current knowledge and identify promising strategies to pursue in future preclinical research directed at SCI repair. Moving forward, it is important to note that the majority of included studies carried out implantations of biomaterials at an acute phase following SCI. It is imperative that researchers adopt appropriate in vivo models at sub-acute and chronic stages to assess biomaterial-based combination strategies at clinically relevant time points. Finally, biomaterial suitability for SCI repair should be assessed using in vitro and/or ex vivo models before advancing to in vivo testing, to minimise the likelihood of a major animal welfare concern. (1343)

## Supplementary information

Supplementary Materials

## Data Availability

The statistical analysis code and datasets analysed during this study are openly available from the Open Science Framework at https://osf.io/bgw73/.
