# Supplementary material for: Effectiveness of biomaterial-based combination strategies for spinal cord repair – a systematic review and meta-analysis of preclinical literature
Source: Spinal Cord. 2022 May 23;60(12):1041–9. doi: 10.1038/s41393-022-00811-z (PMC9712119; doi:10.1038/s41393-022-00811-z)
Supplement: Supplementary file 1 — Supplementary Materials [file 41393_2022_811_MOESM1_ESM.docx]

**Supplementary materials**

**List of eFigures and eTables**

eTable 1: Study characteristics of included papers

eFigure 1: Flowchart of study selection and analysis process.

eTable 2: Meta-regression analysis on effect of biomaterial specific outcomes in SCI regeneration conducted on grouped outcomes for biocompatibility related secondary outcomes *in vitro* and *in vivo*.

eFigure 2: Different effects of biomaterials alone on locomotor and *in vivo* axonal regeneration outcomes.

eTable 3: Multivariable analysis

eTable 4: Meta-regression analysis on effect of biomaterial specific outcomes in SCI regeneration conducted on grouped outcomes for biocompatibility related secondary outcomes *in vitro* and *in vivo*.

eFigure 3: Different effects of biomaterials-based combination strategies on locomotor outcomes

eFigure 4: Different effects of biomaterials-based combination strategies on axonal regeneration outcomes

eFigure 5: Differential effects of study quality on locomotor outcomes

eFigure 6: Differential effects of study design on locomotor outcomes.

eFigure 7: Different effect sizes of biomaterials-based combinations on locomotor and axonal regeneration outcomes.

eFigure 8: Influence of the treatment strategy on locomotor recovery outcomes.

**Online-only methods**

**Protocol deviations**

Our study protocol was initially published in September 2016 on the CAMARADES website (<https://drive.google.com/file/d/0B5x-sP1A05kgMkFqM3VjMkF1OWs/view>). Our first search was performed in September 2016. However, due to the length of the data extraction process, we updated our database to include more recent studies published up to 04/2018. The protocol file was therefore updated (dated 04/2018) accordingly to create the final version (<https://drive.google.com/file/d/1bCHo6bvHOx8GCwLjVvkMNoOedHCq0Wl9/view>).

Deviations from the updated 2018 protocol are listed below:

| **Item in 2018 protocol** | **Protocol deviation and justification** |
| --- | --- |
| **Author list** | **Ezgi Tanriver-Ayder, Emily Sena, Catriona Cunningham and Malcolm Macleod were added. Ruben Rodrigues Machado removed.** |
| **Risk of bias checklist included “(i) publication in peer-reviewed journal”** | **This item was changed to “reporting of animal exclusions“.** All publications included in this study were published in peer-review journals. We added ‘’ reporting of animal exclusion’’ as it has been identified as important to optimize the predictive value of preclinical research  ([Landis, Amara et al. 2012](#_ENREF_64)). |
| A checklist for assessing overall quality of study design in Objective 1:   - “(i) testing of mechanical properties (1); - (ii) investigation of host immune response (1); - (iii) study of cellular response with cell lines (1) or primary cells (2); - (iv) assessment of biomaterial’s drug release ability (1).   Quality scores will be matched to outcome measurements results; this will help determine whether assessing all the biomaterial properties characteristics has an effect on the overall success of the combination strategy.” | We planned to examine outcomes relating to axonal regeneration *in vitro* and to biocompatibility properties (i.e. immune response, cell viability, cell migration, cell differentiation etc.) as a measure of study design quality. However, there were too few studies reporting these variables, making it impossible to proceed with this method of assessment. |
| Objective 3.3. Quantitative analysis of publication bias, study design and study quality. | We did not perform publication bias, study design or study quality analysis for Objective 3 as studies in Objective 3 were already included in the dataset for Objective 2. |

**Inclusion criteria**

Following the database search, full text publications were selected based on the following inclusion criteria:

▪ Types of studies – animal models of SCI where the use of a biomaterial and at least one more regeneration strategy is tested (i.e. growth factor or cell transplantation).

▪ *In vitro* models for SCI where primary CNS cells, neuronal cell lines or tissue slices are used.

▪ *In vivo* models for SCI (transection model, contusion model, compression model).

**Exclusion criteria**

▪ Studies that do not include the use of a biomaterial with at least one more strategy (i.e. growth factor, cell therapy and/or protein overexpression)

▪ Any human studies or clinical trials

▪ Studies that target neurodegenerative diseases (i.e. Alzheimer’s disease, Parkinson’s disease, motor neuron diseases, Spinal muscular atrophy, spinal root avulsion)

▪ *In vivo* studies that only reported qualitative behavioural or histological or electrophysiology results

▪ All publications that do not present results compared to a suitable control. The suitable control for *in vivo* studies is intended as SCI only condition where no treatment has been added (i.e. no biomaterial or growth factor or cell therapy). The suitable control for *in vitro* studies is intended as cells only condition where no treatment has been added (i.e. cells are not cultured in the presence of a biomaterial or growth factor/drug).

▪ All publications/outcome measurements that do not state n-numbers or a measure of variance e.g. SD. SEM.

**List of primary and secondary outcomes and comparisons of Primary outcomes *in vivo***

- locomotor recovery (**NMD**) included SCI-related behavioural outcomes e.g. Basso, Beattie and Bresnahan (BBB), Basso Mouse Scale (BMS), and Olby locomotor score.
- Axonal Regeneration *in vivo* (**SMD-1**) included SCI-related histological outcomes.
- **Primary outcome *in vitro***:
- Axonal regeneration *in vitro* (**SMD-2**)
- **Secondary outcome**s grouped as (**SMD-3**):
- expression of inflammatory marker (histological)
- expression of inflammatory marker (molecular)
- cell adhesion
- cell viability
- cell migration
- cell proliferation
- cell differentiation

Where different measures of behavioural outcome were reported from the same cohort of animals at the same time point, effect size measures were first combined using fixed-effect meta-analysis (nesting) followed by a random-effects model ([Vesterinen, Sena et al. 2014](#_ENREF_120)).

If a time range was given, the latest time point was used. For meta-analysis, the control group’s sample size was adjusted according to the number of treatment groups it served ([Vesterinen, Sena et al. 2014](#_ENREF_120)).

Graphical outcome data were extracted using Acrobat Reader DC ruler tool.

**Online-only results – Table and Figures:**

**eTable 1: Study characteristics of included papers**

| **Author** | **Year** | **Biomaterial name** | **Biomaterial type** | **Animal** | **Type of Injury** | **Level** | **Drug** | **Stem Cells** | **References** |
| --- | --- | --- | --- | --- | --- | --- | --- | --- | --- |
| Altinova | 2016 | Collagen | Natural | Rat | Transection | C3-C4 |  | OECs | ([Altinova, Mollers et al. 2016](#_ENREF_1)) |
| Anderson | 2016 | Diblock co-polypeptide hydrogel | Synthetic | Mouse | Compression | T10 | NT3 and BDNF |  | ([Anderson, Burda et al. 2016](#_ENREF_2)) |
| Ansorena | 2013 | Alginate-PLGA | Natural and Synthetic | Rat | Hemisection | T9-T10 | GDNF |  | ([Ansorena, De Berdt et al. 2013](#_ENREF_3)) |
| Asmani | 2013 | Fibrin | Natural |  |  |  | FGF-2, EGF and PDGF-AA | OPCs | ([Asmani, Ai et al. 2013](#_ENREF_4)) |
| Baumann | 2010 | HAMC-PLGA | Natural and Synthetic | Rat | Compression | T2 |  |  | ([Baumann, Kang et al. 2010](#_ENREF_7)) |
| Baumann | 2009 | HAMC | Synthetic |  |  |  |  |  | ([Baumann, Kang et al. 2009](#_ENREF_6)) |
| Berns | 2017 | SAPs | Synthetic |  |  |  | Tenascin-C |  | ([Berns, Alvarez et al. 2016](#_ENREF_8)) |
| Bozkurt | 2010 | Chitosan | Natural | Rat | Compression | T7-T9 |  | NSCs | ([Bozkurt, Mothe et al. 2010](#_ENREF_9)) |
| Breen | 2016 | Collagen | Natural | Rat | Hemisection | T9-T10 | NT3 |  | ([Breen, Kraskiewicz et al. 2017](#_ENREF_10)) |
| Burdick | 2005 | PEG | Synthetic |  |  |  | CNTF |  | ([Burdick, Ward et al. 2006](#_ENREF_11)) |
| Buzoianu-Anguiano | 2015 | Fibrin | Natural | Rat | Transection | T9 |  | bMSCs | ([Buzoianu-Anguiano, Orozco-Suarez et al. 2015](#_ENREF_12)) |
| Caron | 2016 | Composite hydrogel + RGD | Natural and Synthetic | Mouse | Compression | T12 | ECM | hMSCs | ([Caron, Rossi et al. 2016](#_ENREF_13)) |
| Chan | 2014 | PHB-b-DEG | Synthetic |  | Compression | T2 |  |  | ([Chan, Russell et al. 2014](#_ENREF_14)) |
| Chen | 2015 | HEMA-MOETACL Hydrogel | Synthetic | Rat | Transection | T9 | bFGF |  | ([Chen, He et al. 2015](#_ENREF_15)) |
| Chen | 2014 | Acellular spinal cord | Natural | Rat | Hemisection | T9-T10 |  | bMSCs | ([Chen, Zhang et al. 2014](#_ENREF_16)) |
| Chen | 2011 | Chitosan | Natural | Rat | Transection | T8-T10 |  | bMSCs | ([Chen, Yang et al. 2011](#_ENREF_17)) |
| Cholas | 2012 | Collagen | Natural | Rat | Hemisection | T8-T9 | ChABC and antiNgR | MSCs | ([Cholas, Hsu et al. 2012](#_ENREF_18)) |
| Cholas | 2012 | Collagen | Natural | Rat | Hemisection | T8-T9 | ChABC and antiNgR | MSCs | ([Cholas, Hsu et al. 2012](#_ENREF_19)) |
| Cigognini | 2011 | SAPs | Synthetic | Rat | Contusion | T9-10 | BMHP1 |  | ([Cigognini, Satta et al. 2011](#_ENREF_20)) |
| De Berdt | 2015 | Fibrin | Natural |  | Hemisection | T9 |  | APSCs | ([De Berdt, Vanacker et al. 2015](#_ENREF_21)) |
| DePaul | 2015 | Fibrin | Natural | mouse | Transection | T8 | aFGF |  | ([DePaul, Lin et al. 2015](#_ENREF_22)) |
| des Rieux | 2013 | Alginate | Natural | Rat | Hemisection | T9-T10 | VEGF |  | ([des Rieux, De Berdt et al. 2014](#_ENREF_23)) |
| Deumens | 2012 | Collagen | Natural | Rat | Hemisection | T13 |  | OECs | ([Deumens, Van Gorp et al. 2013](#_ENREF_24)) |
| Donaghue | 2015 | PLGA | Synthetic | Rat | Compression | T1-T2 | NT3 and antiNgR |  | ([Elliott Donaghue, Tator et al. 2016](#_ENREF_27)) |
| Downing | 2012 | PLLA | Synthetic | Rat | Hemisection | C4-C6 | Rolipram |  | ([Downing, Wang et al. 2012](#_ENREF_25)) |
| Elias | 2015 | OEGMA | Synthetic |  |  |  |  |  | ([Elias, Liu et al. 2015](#_ENREF_26)) |
| Erdogan | 2010 | Alginate | Natural | Rat | Hemisection | T8-T9 |  | rfUCSCs | ([Erdogan, Bavbek et al. 2010](#_ENREF_28)) |
| Fan | 2010 | Collagen | Natural | Rat | Hemisection | T8-T10 | NT3 |  | ([Fan, Xiao et al. 2010](#_ENREF_30)) |
| Fan | 2011 | PLGA | Synthetic | Rat | Transection | T8-T10 | NT3 |  | ([Fan, Zhang et al. 2011](#_ENREF_31)) |
| Fan | 2017 | Collagen | Natural | Rat | Transection | T8 | EGFR AB |  | ([Fan, Li et al. 2017](#_ENREF_29)) |
| Ferrero-Gutierrez | 2013 | Albumin | Natural | Rat | Transection | T7 |  | ADSCs and OECs | ([Ferrero-Gutierrez, Menendez-Menendez et al. 2013](#_ENREF_32)) |
| Fouad | 2005 | Matrigel | Natural | Rat | Transection | T8 | ChABC | Schwann cells | ([Fouad, Schnell et al. 2005](#_ENREF_33)) |
| Francis | 2017 | Chitosan-Alginate | Natural |  |  |  | NT3 and ChABC |  | ([Francis, Hunger et al. 2017](#_ENREF_34)) |
| Fuhrmann | 2015 | HA | Natural | Rat | Compression | T2 |  |  | ([Fuhrmann, Obermeyer et al. 2015](#_ENREF_35)) |
| Fuhrmann | 2016 | HAMC-RGD | Synthetic | Rat | Compression | T2 | PDGF-A | OPCs | ([Fuhrmann, Tam et al. 2016](#_ENREF_36)) |
| Ganz | 2017 | PLLA-PLGA | Synthetic | Rat | Transection | T10 | FGF-2 and EGF | hOMSCs | ([Ganz, Shor et al. 2017](#_ENREF_37)) |
| Gelain | 2012 | SAPs | Synthetic | Rat | Contusion | T9-T10 | FAQ |  | ([Gelain, Cigognini et al. 2012](#_ENREF_38)) |
| Gomes | 2016 | GG-GRGDS | Natural and Synthetic | Rat | Hemisection | L1 |  | ADSCs and OECs | ([Gomes, Mendes et al. 2016](#_ENREF_39)) |
| Grulova | 2015 | Alginate | Natural | Rat | Compression | T8-T9 | bFGF and EGF |  | ([Grulova, Slovinska et al. 2015](#_ENREF_40)) |
| Gunther | 2015 | Alginate | Natural | Rat | Hemisection | C5 | BDNF | bMSCs | ([Gunther, Weidner et al. 2015](#_ENREF_41)) |
| Gupta | 2006 | HAMC | Natural | Rat | Compression | T2 |  |  | ([Gupta, Tator et al. 2006](#_ENREF_42)) |
| Hakim | 2015 | OPF | Synthetic | Rat | Transection | T9-T10 |  | Schwann cells | ([Hakim, Esmaeili Rad et al. 2015](#_ENREF_44)) |
| Han | 2010 | LOCS | Natural | Rat | Transection | T8-T9 | BDNF |  | ([Han, Jin et al. 2010](#_ENREF_45)) |
| Han | 2009 | Collagen | Natural | Rat | Hemisection | T8-10 | BDNF |  | ([Han, Sun et al. 2009](#_ENREF_46)) |
| Han | 2015 | LOCS | Natural | Dog | Transection | T12 | BDNF |  | ([Han, Wang et al. 2015](#_ENREF_48)) |
| Han | 2014 | Collagen | Natural | Dog | Transection | T12 | BDNF |  | ([Han, Wang et al. 2014](#_ENREF_47)) |
| He | 2009 | Chitosan | Natural |  |  |  |  |  | ([He, Zhang et al. 2009](#_ENREF_49)) |
| Hejcl | 2010 | HPMA-RGD | Synthetic | Rat | Compression | T7-T10 | RGD | MSCs | ([Hejcl, Sedy et al. 2010](#_ENREF_50)) |
| Hou | 2012 | SAPs | Synthetic | Rat | Hemisection | T12-L1 |  | Motoneurons | ([Hou, Wu et al. 2012](#_ENREF_51)) |
| Hsueh | 2012 | Chitosan | Natural |  |  |  |  | hADSC | ([Hsueh, Chiang et al. 2012](#_ENREF_52)) |
| Hwang | 2011 | PCL | Synthetic | Rat | Hemisection | T7-T8 | NT3 and ChABC | NSCs | ([Hwang, Kim et al. 2011](#_ENREF_53)) |
| Itosaka | 2009 | Fibrin | Natural | Rat | Hemisection | T8 |  | bMSCs | ([Itosaka, Kuroda et al. 2009](#_ENREF_54)) |
| Kueh | 2012 | PLGA | Synthetic |  |  |  |  | OECs | ([Kueh, Li et al. 2012](#_ENREF_62)) |
| Jian | 2015 | Chitosan | Natural | Rat | Hemisection | T8 | SB216763 | NSCs | ([Jian, Yixu et al. 2015](#_ENREF_56)) |
| Jiao | 2017 | Silk-Alginate | Natural | Rat | Contusion | T9 | GDNF | hUCMSCs | ([Jiao, Lou et al. 2017](#_ENREF_57)) |
| Johnson | 2010 | Fibrin | Natural | Rat | Hemisection | T9 | NT3 |  | ([Johnson, Parker et al. 2009](#_ENREF_58)) |
| Kabiri | 2015 | PLLA-SWCNT | Synthetic | Rat |  |  |  | OECs | ([Kabiri, Oraee-Yazdani et al. 2015](#_ENREF_59)) |
| Kang | 2013 | HAMC-PLGA | Natural and Synthetic | Rat | Compression | T2 | FGF-2 |  | ([Kang, Baumann et al. 2013](#_ENREF_60)) |
| Kim | 2011 | PLGA-Chitosan | Natural and Synthetic | Rat | Transection | T8 | dbcAMP | NSPCs | ([Kim, Zahir et al. 2011](#_ENREF_61)) |
| Lee | 2002 | Fibrin | Natural | Rat | Transection | T8 | aFGF |  | ([Lee, Hsiao et al. 2002](#_ENREF_65)) |
| Lee | 2007 | Fibrin | Natural | Rat | Transection | T8 | aFGF |  | ([Lee, Lin et al. 2007](#_ENREF_66)) |
| Li | 2013 | LOCS | Natural | Rat | Hemisection | T13-L2 | Cetuximab |  | ([Li, Xiao et al. 2013](#_ENREF_69)) |
| Li | 2016 | Gelatin | Natural | Rat | Transection | T10 |  | MScs | ([Li, Che et al. 2016](#_ENREF_67)) |
| Li | 2016 | Collagen | Natural | Rat | Transection | T9 | BDNF and NT-3 |  | ([Li, Han et al. 2016](#_ENREF_68)) |
| Li | 2017 | LOCS | Natural | Dog | Transection | T8 | Cetuximab |  | ([Li, Zhao et al. 2017](#_ENREF_70)) |
| Lindsey | 2015 | MAX8-F-moc gel | Synthetic |  |  |  | NGF |  | ([Lindsey, Piatt et al. 2015](#_ENREF_71)) |
| Liu | 2013 | Fibrin | Natural | Rat | Transection | T10 |  | MSCs | ([Liu, Chen et al. 2013](#_ENREF_73)) |
| Liu | 2015 | PLGA-PEG | Synthetic | Rat | Transection | T10-T11 |  | NSCs | ([Liu, Huang et al. 2015](#_ENREF_72)) |
| Lu | 2012 | Fibrin | Natural | Rat | Transection | T3 |  | NSCs | ([Lu, Wang et al. 2012](#_ENREF_74)) |
| Macaya | 2013 | Collagen | Natural |  |  |  | FGF-2 |  | ([Macaya, Hayakawa et al. 2013](#_ENREF_75)) |
| Milbreta | 2016 | PCLEEP | Synthetic | Rat | Hemisection | C5 | NT3 |  | ([Milbreta, Nguyen et al. 2016](#_ENREF_76)) |
| Nguyen | 2016 | Sodium alginate-gelatine | Natural |  |  |  | Fucoidan |  | ([Nguyen, Ko et al. 2016](#_ENREF_77)) |
| Ni | 2015 | Chitosan | Natural and Synthetic | Rat | Hemisection | T7-T9 | ChABC |  | ([Ni, Xia et al. 2015](#_ENREF_78)) |
| Nomura | 2006 | PHEMA-MMA | Natural and Synthetic | Rat | Transection | T6-T10 | aFGF |  | ([Nomura, Katayama et al. 2006](#_ENREF_80)) |
| Nomura | 2008 | Chitosan | Natural | Rat | Hemisection | T7-T9 |  |  | ([Nomura, Zahir et al. 2008](#_ENREF_81)) |
| Nomura | 2008 | Chitosan | Natural | Rat | Transection | T8 |  | NSCs | ([Nomura, Baladie et al. 2008](#_ENREF_79)) |
| Nothias | 2005 | Collagen | Natural | Rat | Transection | T8-T9 | Exercise | Fibroblasts | ([Nothias, Mitsui et al. 2005](#_ENREF_82)) |
| Pakulska | 2017 | Methylcellulose-PLGA | Natural and Synthetic | Rat | Compression | T1-T2 | ChABC |  | ([Pakulska, Tator et al. 2017](#_ENREF_83)) |
| Pal | 2013 | ION-agarose | Natural and Synthetic | Rat | Transection | T11 | Magnetic field |  | ([Pal, Singh et al. 2013](#_ENREF_84)) |
| Park | 2010 | HA | Natural | Rat | Compression | T10 | BDNF | hMSCs | ([Park, Lim et al. 2010](#_ENREF_85)) |
| Park | 2013 | Alginate | Natural | Rat | Contusion | T7 | Wnt-3 | Fibroblasts | ([Park, Min et al. 2013](#_ENREF_86)) |
| Park | 2012 | Matrigel | Natural | Dog | Compression | L1 |  | MSCs | ([Park, Lee et al. 2012](#_ENREF_87)) |
| Patel | 2010 | Matrigel | Natural | Rat | Contusion | T8 |  | Schwann cells | ([Patel, Joseph et al. 2010](#_ENREF_88)) |
| Peng | 2017 | Collagen | Natural | Rat | Hemisection | T9 |  | MSCs | ([Peng, Gao et al. 2016](#_ENREF_89)) |
| Pertici | 2013 | PHPMA | Synthetic | Rat | Hemisection | T10 |  |  | ([Pertici, Amendola et al. 2013](#_ENREF_90)) |
| Pertici | 2014 | PLA-b-PHEMA | Synthetic | Rat | Hemisection | C6-T13 |  |  | ([Pertici, Trimaille et al. 2014](#_ENREF_91)) |
| Pritchard | 2010 | PLGA | Synthetic | Monkey | Hemisection | T9-T10 |  | hNSCs | ([Pritchard, Slotkin et al. 2010](#_ENREF_92)) |
| Rad | 2014 | PEG | Synthetic | Rat |  |  |  |  | ([Rad, Mobasheri et al. 2014](#_ENREF_93)) |
| Rauch | 2009 | PLGA-PEG/PLL | Synthetic | Rat | Hemisection | T9-T10 |  | NSCs and ECs | ([Rauch, Hynes et al. 2009](#_ENREF_95)) |
| Raynald | 2015 | HA | Natural | Rat | Hemisection | T9 |  | bMSCs | ([Raynald, Li et al. 2016](#_ENREF_96)) |
| Ribeiro-Samy | 2013 | PHB-HV | Synthetic | Rat | Hemisection | T8-T9 |  |  | ([Ribeiro-Samy, Silva et al. 2013](#_ENREF_97)) |
| Rooney | 2011 | OPF | Synthetic | Rat | Transection | T8-T9 | dbcAMP | MSCs and SCs | ([Rooney, Knight et al. 2011](#_ENREF_98)) |
| Ropper | 2017 | PLGA | Synthetic | Rat | Hemisection | T9-T10 | FGF | hMSCs | ([Ropper, Thakor et al. 2017](#_ENREF_99)) |
| Ruzicka | 2013 | PHEMA | Synthetic | Rat | Hemisection | T8 | Serotonine | hfNSCs | ([Ruzicka, Romanyuk et al. 2013](#_ENREF_100)) |
| Shanbhag | 2010 | Alginate | Natural |  |  |  | BDNF and NGF | Fibroblasts | ([Shanbhag, Lathia et al. 2010](#_ENREF_102)) |
| Sharp | 2014 | Fibrin | Natural | Rat | Transection | T3 | BDNF, NT-3, MDL28170, PDGF-AA and IGF-1 | NSCs | ([Sharp, Yee et al. 2014](#_ENREF_103)) |
| Shen | 2010 | Silk | Natural | Rat |  |  |  | OECs | ([Shen, Qian et al. 2010](#_ENREF_104)) |
| Shi | 2014 | Collagen | Natural | Rat | Hemisection | T9 | bFGF |  | ([Shi, Gao et al. 2014](#_ENREF_105)) |
| Silva | 2013 | Gellan gum | Natural |  |  |  | GRGDS | MSCs | ([Silva, Moreira et al. 2013](#_ENREF_106)) |
| Stokols | 2004 | Agarose | Natural |  |  |  | NGF |  | ([Stokols and Tuszynski 2004](#_ENREF_108)) |
| Sun | 2017 | Chitosan | Natural | Mouse | Hemisection | T9 | NT3 | hUCMSCs | ([Sun, Shao et al. 2017](#_ENREF_109)) |
| Tang | 2010 | Collagen | Natural |  |  |  |  |  | ([Tang, Liu et al. 2010](#_ENREF_110)) |
| Tavakol | 2014 | Matrigel | Natural | Rat | Compression | T10 |  | hEnSCs | ([Tavakol, Aligholi et al. 2014](#_ENREF_111)) |
| Taylor | 2004 | Fibrin | Natural |  |  |  | NT3 |  | ([Taylor, McDonald et al. 2004](#_ENREF_112)) |
| Taylor | 2006 | Fibrin | Natural | Rat | Transection | T9 | NT3 |  | ([Taylor, Rosenzweig et al. 2006](#_ENREF_113)) |
| Teng | 2002 | PLGA-PDL | Synthetic | Rat | Hemisection | T9-T10 |  | NSCs | ([Teng, Lavik et al. 2002](#_ENREF_114)) |
| Terraf | 2017 | PCL | Synthetic | Rat | Hemisection | T9 | Crocin | hEnSCs | ([Terraf, Kouhsari et al. 2017](#_ENREF_115)) |
| Tsai | 2005 | Fibrin | Natural | Rat | Transection | T8 | FGF-1 |  | ([Tsai, Krassioukov et al. 2005](#_ENREF_117)) |
| Tsai | 2006 | PHEMA-MMA-Fibrin | Natural and Synthetic | Rat | Transection | T8 | FGF-1 |  | ([Tsai, Dalton et al. 2006](#_ENREF_116)) |
| Tukmachev | 2016 | ECM | Natural | Rat | Hemisection | T8 |  | MSCs | ([Tukmachev, Forostyak et al. 2016](#_ENREF_118)) |
| Wang | 2011 | Collagen-HSPG | Natural |  |  |  |  | NSCs | ([Wang, Zhou et al. 2011](#_ENREF_126)) |
| Wang | 2007 | Collagen | Natural |  |  |  |  | ADSCs and OECs | ([Wang, Han et al. 2007](#_ENREF_121)) |
| Wang | 2013 | Chitosan | Natural | Rat | Transection | T8 | NT3 |  | ([Wang, Li et al. 2013](#_ENREF_125)) |
| Wang | 2012 | Alginate | Natural | Rat | Transection | T8-T9 |  | Schwann cells | ([Wang, Liu et al. 2012](#_ENREF_122)) |
| Wang | 2017 | Collagen | Natural | Rat | Transection | T8-T9 |  | hUCMSCs | ([Wang, Xiao et al. 2017](#_ENREF_123)) |
| Wang | 2014 | Sodium hyaluronate | Natural | Rat | Transection | T8-T9 | CNTF |  | ([Wang, Zhang et al. 2014](#_ENREF_124)) |
| Wei | 2010 | HA-PLL | Natural and Synthetic | Rat | Hemisection | T8-T9 | antiNgR |  | ([Wei, He et al. 2010](#_ENREF_127)) |
| Wen | 2016 | HA-PLGA | Natural and Synthetic | Rat | Hemisection | T9-T10 | BDNF and VEGF |  | ([Wen, Yu et al. 2016](#_ENREF_128)) |
| Wen | 2016 | HA-PLGA | Natural and Synthetic | Rat | Hemisection | T9-T10 | antiNgR |  | ([Wen, Yu et al. 2016](#_ENREF_128)) |
| Wilems | 2015 | Fibrin-PLGA | Natural and Synthetic | Rat | Hemisection | T8 | ChABC and NEP1-40 |  | ([Wilems, Pardieck et al. 2015](#_ENREF_130)) |
| Wu | 2013 | PF-127 | Synthetic | Rat | Transection | T10 | Lingo-1 |  | ([Wu, Cen et al. 2013](#_ENREF_131)) |
| Wu | 2014 | Chitosan | Natural | Rat | Contusion | T10 | Ferulic acid |  | ([Wu, Lee et al. 2014](#_ENREF_132)) |
| Xiong | 2009 | PLGA | Synthetic |  |  |  | NT3 | NSCs | ([Xiong, Zeng et al. 2009](#_ENREF_133)) |
| Xue | 2015 | Chitin | Natural | Rat | Hemisection | T8 |  | MSCs | ([Xue, Wu et al. 2015](#_ENREF_134)) |
| Yang | 2005 | PLG | Synthetic |  |  |  | NGF |  | ([Yang, De Laporte et al. 2005](#_ENREF_136)) |
| Yang | 2015 | Chitosan | Natural | Rat | Transection | T7-T8 | NT3 |  | ([Yang, Zhang et al. 2015](#_ENREF_138)) |
| Yang | 2017 | PLGA | Synthetic | Rat | Transection | T9 |  | Activated Schwann cells and MSCs | ([Yang, Zhang et al. 2017](#_ENREF_135)) |
| Yang | 2015 | PEGDM | Synthetic |  |  |  | IKVAV |  | ([Yang, Khan et al. 2015](#_ENREF_137)) |
| Zaminy | 2013 | Collagen | Natural | Rat | Hemisection | T9-T10 |  | ADSCs | ([Zaminy, Shokrgozar et al. 2013](#_ENREF_139)) |
| Zaminy | 2013 | Collagen | Natural | Rat | Hemisection | T9 |  | MSCs | ([Zaminy, Shokrgozar et al. 2013](#_ENREF_140)) |
| Zhang | 2009 | Fibrin | Natural | Rat | Transection | T9 | BDNF | hMSCs | ([Zhang, Zhang et al. 2009](#_ENREF_142)) |
| Zhang | 2016 | Chitosan | Natural | Rat | Contusion | T9 |  | DPSCs | ([Zhang, Lu et al. 2016](#_ENREF_141)) |
| Zhao | 2016 | Heparin-poloxamer | Natural and Synthetic | Rat | Contusion | T9 | NGF |  | ([Zhao, Jiang et al. 2016](#_ENREF_144)) |
| Zhao | 2017 | Heparin-poloxamer | Natural and Synthetic | Rat | Compression | T9 | GDNF |  | ([Zhao, Jiang et al. 2017](#_ENREF_143)) |

**eTable 2: Meta-regression analysis on effect of biomaterial specific outcomes in SCI regeneration conducted on grouped outcomes for biocompatibility related secondary outcomes *in vitro* and *in vivo*.**

| Improvement in biocompatibility-related outcomes for in vitro and in vivo | | | | |
| --- | --- | --- | --- | --- |
| **Biomaterial name** | **Effect size (SD)** | **P>\|t\|** | **95% CI** | **Frequency (%)** |
| Matrigel | 1.8 | 0.167 | [-0.6 to 4.1] | 6.9 |
| Fibrin | 1.4 | 0.262 | [-0.9 to 3.8] | 8.62 |
| Chitosan | 1.0 | 0.419 | [-1.2 to 3.2] | 10.34 |
| HA-PLGA | 0.8 | 0.582 | [-1.7 to 3.3] | 5.17 |
| Heparin-poloxamer | 0.6 | 0.688 | [-2 to 3.2] | 5.17 |
| Collagen | 0.1 | 0.886 | [-1.4 to 1.6] | 12.07 |
| Other | 0.0 | 0.862 | [-1.7 to 1.6] | 37.93 |
| Alginate | -0.4 | 0.656 | [-2.4 to 1.7] | 8.62 |
| Silk | -0.4 | 0.694 | [-3.1 to -2.3] | 5.17 |
|  | comparisons=58, p=0.5457, Tau^2^= 1.558, I^2^=65%, adj R^2^= -11.6% | | | |

**eTable 3: Multivariable meta-regression analysis of the effect of biomaterial-specific outcomes in SCI conducted in grouped outcomes for NMD locomotor recovery data and SMD *in vivo* axonal regeneration**

| Multivariate analysis of the effect of biomaterial-specific outcomes on locomotor recovery | | | |
| --- | --- | --- | --- |
| **Biomaterial type/format/name** | **Effect size (%)** | **P>\|t\|** | **95% CI** |
| Collagen Natural Biomaterial in scaffold format | 10.2 | 0.03 | [1.1 to 19.3] |
| Natural and Synthetic | 5.7 | 0.565 | [-8.8 to 20.7] |
| Synthetic | 8.0 | 0.710 | [-3.8 to 19.8] |
| Fiber | 16.5 | 0.714 | [-17.5 to 50.4] |
| Hydrogel | 7.0 | 0.541 | [-3.7 to 17.6] |
| Linear oriented scaffold | 4.6 | 0.247 | [-5 to 14.2] |
| Microsphere loaded hydrogel | 8.5 | 0.823 | [-7.7 to 24.6] |
| Injectable hydrogel | 0.2 | 0.16 | [-13.8 to 14.3] |
| Chitosan | 6.8 | 0.556 | [-5 to 18.5] |
| HA | 12.0 | 0.838 | [-5.2 to 29.2] |
| HA-PLGA | 7.6 | 0.807 | [-13.5 to 28.7] |
| Other | 15.1 | 0.466 | [1.8 to 28.5] |
| PHEMA-MMA | 20.2 | 0.345 | [-0.8 to 41.3] |
| PLGA | 13.4 | 0.701 | [-4.2 to 31.5] |
|  | comparisons=68, p=0.814, Tau^2^= 89.4, I^2^=85.4%, adj R^2^= 0% | | |
| **Multivariate analysis of the effect of biomaterial-specific outcomes on in vivo axonal regeneration** | | | |
| **Biomaterial type/name** | **Effect size (SD)** | **P>\|t\|** | **95% CI** |
| Natural Collagen | 0.78 | 0.075 | [-0.1 to 1.6] |
| Natural and Synthetic | 2.03 | 0.132 | [0.4 to 3.7] |
| Synthetic | 0.84 | 0.921 | [-0.4 to 2.1] |
| Other | 1.23 | 0.434 | [0.1 to 2.4] |
| HA-PLGA | -1.13 | 0.099 | [-3.4 to 1.1] |
| PLGA | 0.81 | 0.6075 | [-0.8 to 2.4] |
|  | comparisons=63, p=0.256, Tau^2^= 1.37, I^2^=76.5%, adj R^2^=0% | | |

**eTable 4: Meta-regression analysis on effect of biomaterial specific outcomes in SCI regeneration conducted on grouped outcomes for SMD Biocompatibility related secondary outcomes *in vitro* and *in vivo.***

| Improvement in biocompatibility related outcomes for in vitro and in vivo | | | | |
| --- | --- | --- | --- | --- |
| **Biomaterial name** | **Effect size (SD)** | **P>\|t\|** | **95% CI** | **Frequency (%)** |
| Heparin-poloxamer + combinations | 7.3 | 0.022 | [1.4 to 13.3] | 4.7 |
| Collagen + combinations | 2.5 | 0.155 | [-0.5 to 5.5] | 11.1 |
| Chitosan + combinations | 1.8 | 0.331 | [-1.1 to 4.9] | 11.1 |
| Fibrin + combinations | 1.8 | 0.350 | [-1.3 to 5.0] | 7.9 |
| Alginate+ combinations | 1.6 | 0.405 | [-1.3 to 4.6] | 6.3 |
| Other + combinations | 1.5 | 0.295 | [-0.6 to 3.7] | 41.2 |
| SAPs + combinations | 1.1 | 0.651 | [-2.2 to 4.4] | 4.7 |
| Fibrin-PLGA + combinations | 0.4 | 0.678 | [-1.5 to 2.2] | 12.7 |
|  | comparisons= 63, p=0.4589, Tau^2^= , I^2^= 70.4%, adj R^2^= -6.54% | | | |


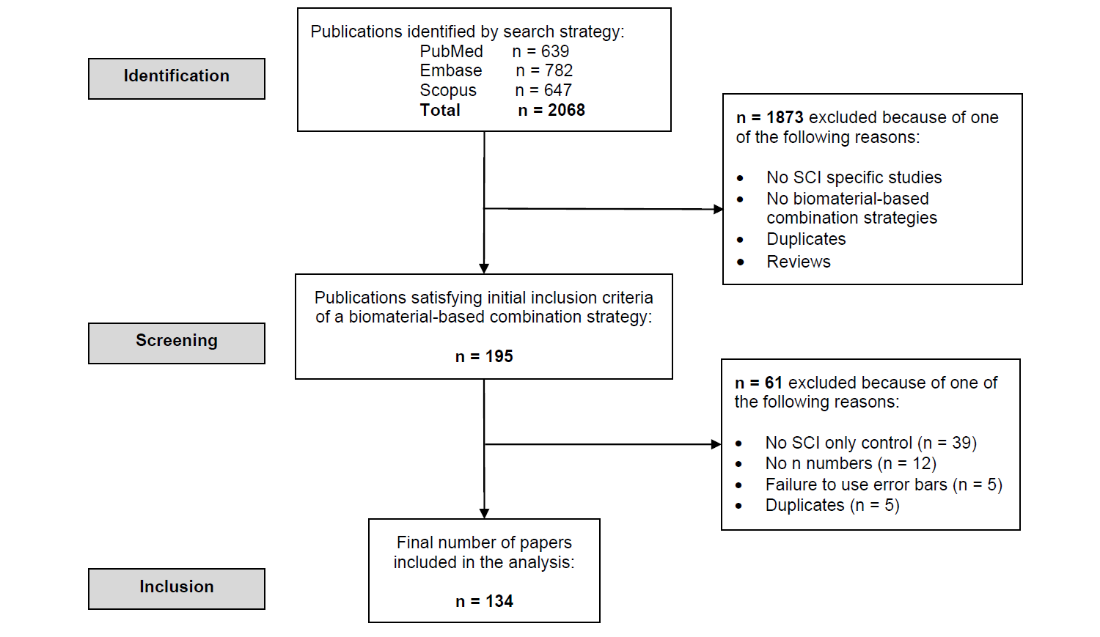


**eFigure 1: Flowchart of study selection and analysis process.**

A systematic search in PubMed, EMBASE and Scopus yielded 2068 unique publications. After application of inclusion and exclusion criteria, data from 134 publications were included in the meta-analysis and study quality/design assessment. Following the data extraction, the analysis was conducted based on the set objectives. Objective 1 includes only comparisons that assessed the effect of biomaterials alone. Objective 2 includes studies that assessed combination strategies *in vitro*, *in vivo*, and/or studied the biomaterial properties. Objective 3 includes studies that carried out investigations only *in vivo*.


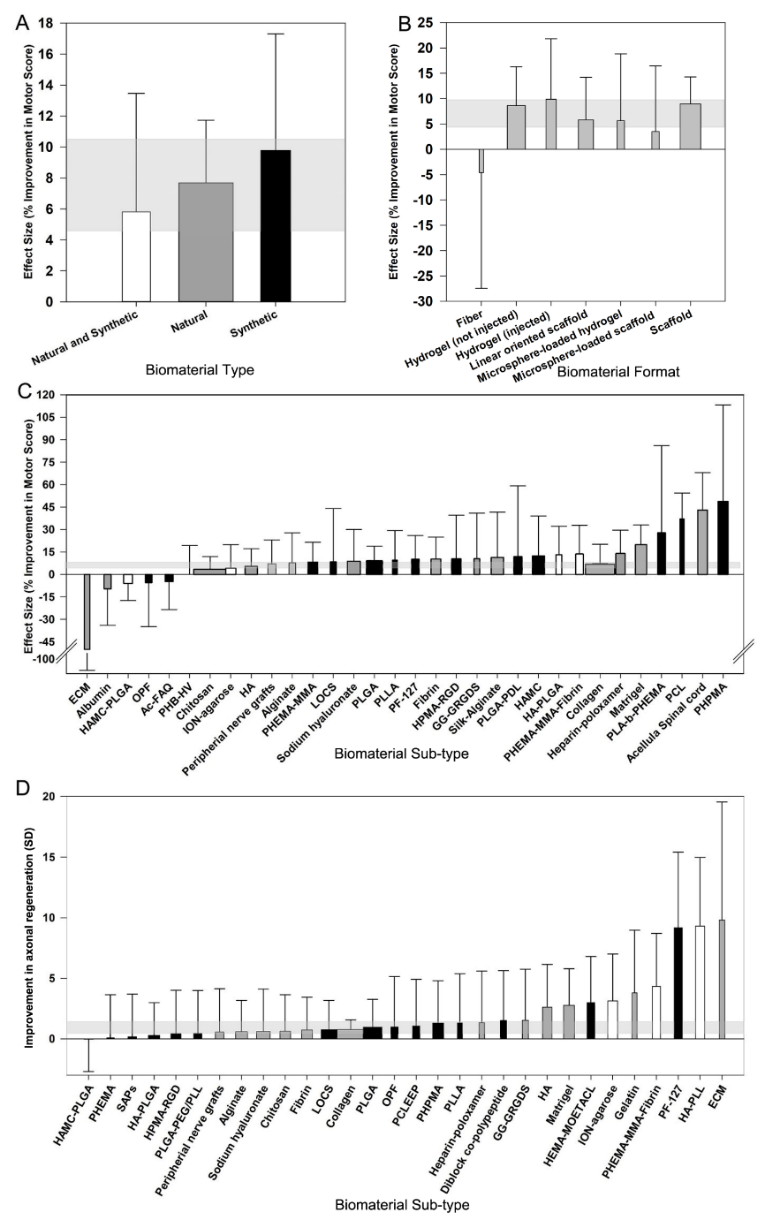


**eFigure 2: Different effects of biomaterials alone on locomotor and *in vivo* axonal regeneration outcomes.**

Effect of the (A) type of biomaterial (natural, synthetic and mixed), (B) biomaterial format or shape, and (C) specific biomaterial on the effect size as a percentage improvement in motor score, and (D) specific biomaterial on the improvement in axonal regeneration in standard deviations (SD). Biomaterials in (C) and (D) are colour coded according to biomaterial types as shown in (A); the bars are white for mixed natural and synthetic biomaterials, grey for natural, and black for synthetic materials. Vertical error bars represent the 95% CI for the individual estimates, and the horizontal shaded grey bar represents the 95% CI of the global estimate. The width of each vertical bar is normalised to the square root of number of animals contributing to that comparison. Number of comparisons for locomotor recovery (C): 68. Number of comparisons for axonal regeneration (D): 63.


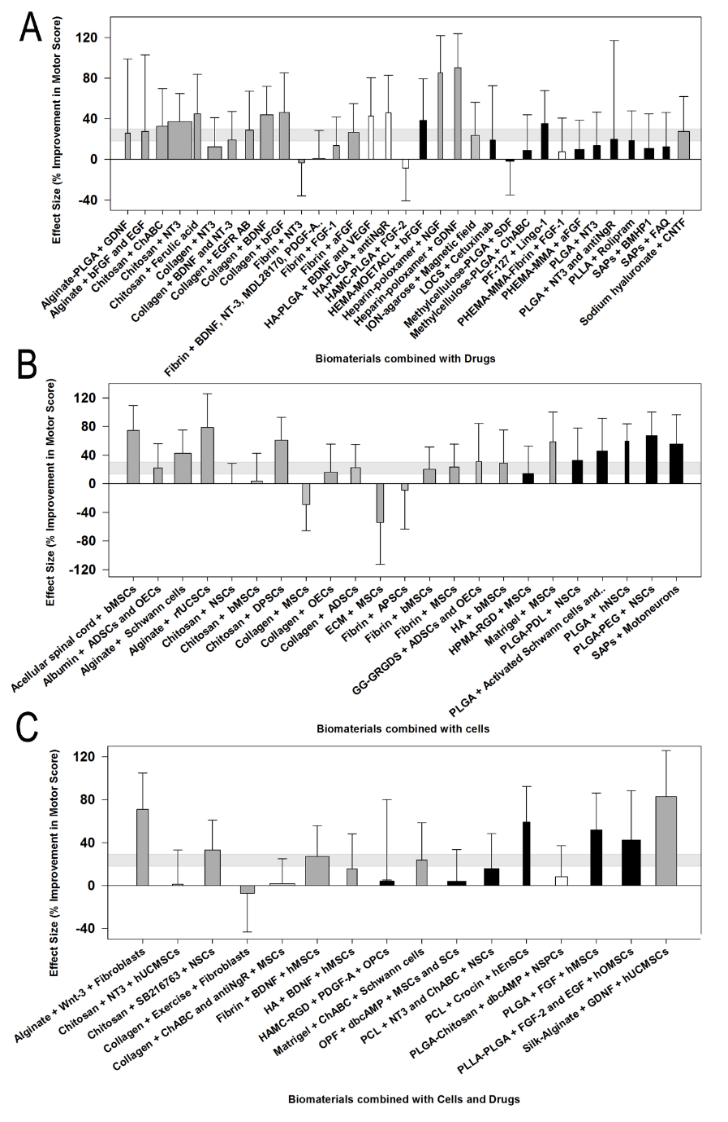


**eFigure 3: Different effects of biomaterial-based combination strategies on locomotor outcomes**.

(A) Effect of the type of biomaterial-based combination on the effect size as a percentage of improvement in motor score on studies performing *in vitro* and/or *in vivo* experiments. Part A shows the biomaterial combined with drugs, B biomaterials combined with cells and C biomaterials combined with cells and drugs. Results are plotted in alphabetical order and variables are colour coded according to the biomaterial type: white for natural and synthetic, grey for natural and black for synthetic. Vertical error bars represent the 95% CI for the individual estimates and the horizontal shaded grey bar represents the 95% CI of the global estimate. The width of each vertical bar is normalised to the square root of the number of animals contributing to that comparison. Number of total comparisons: 102.


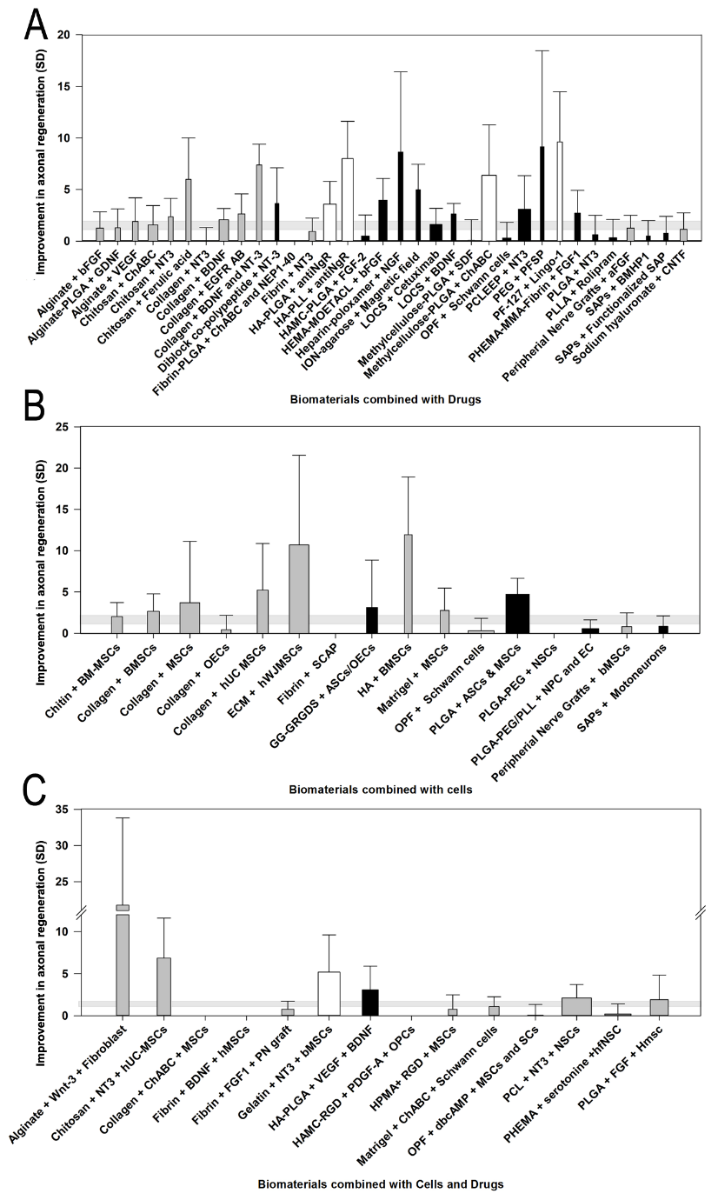


**eFigure 4: Different effects of biomaterials-based combination strategies on axonal regeneration outcomes.**

Effect of the type of biomaterial-based combination on improvement in axonal regeneration (SD) in studies performing *in vitro* and/or *in vivo* experiments. Part A shows the biomaterial combined with drugs, B biomaterials combined with cells and C biomaterials combined with cells and drugs. Results are plotted in alphabetical order and variables are colour coded according to the biomaterial type: white for natural and synthetic, grey for natural and black for synthetic. Vertical error bars represent the 95% CI for the individual estimates and the horizontal shaded grey bar represents the 95% confidence limits of the global estimate. The width of each vertical bar is normalised to the square root of the number of animals contributing to that comparison. Number of total comparisons: 117.


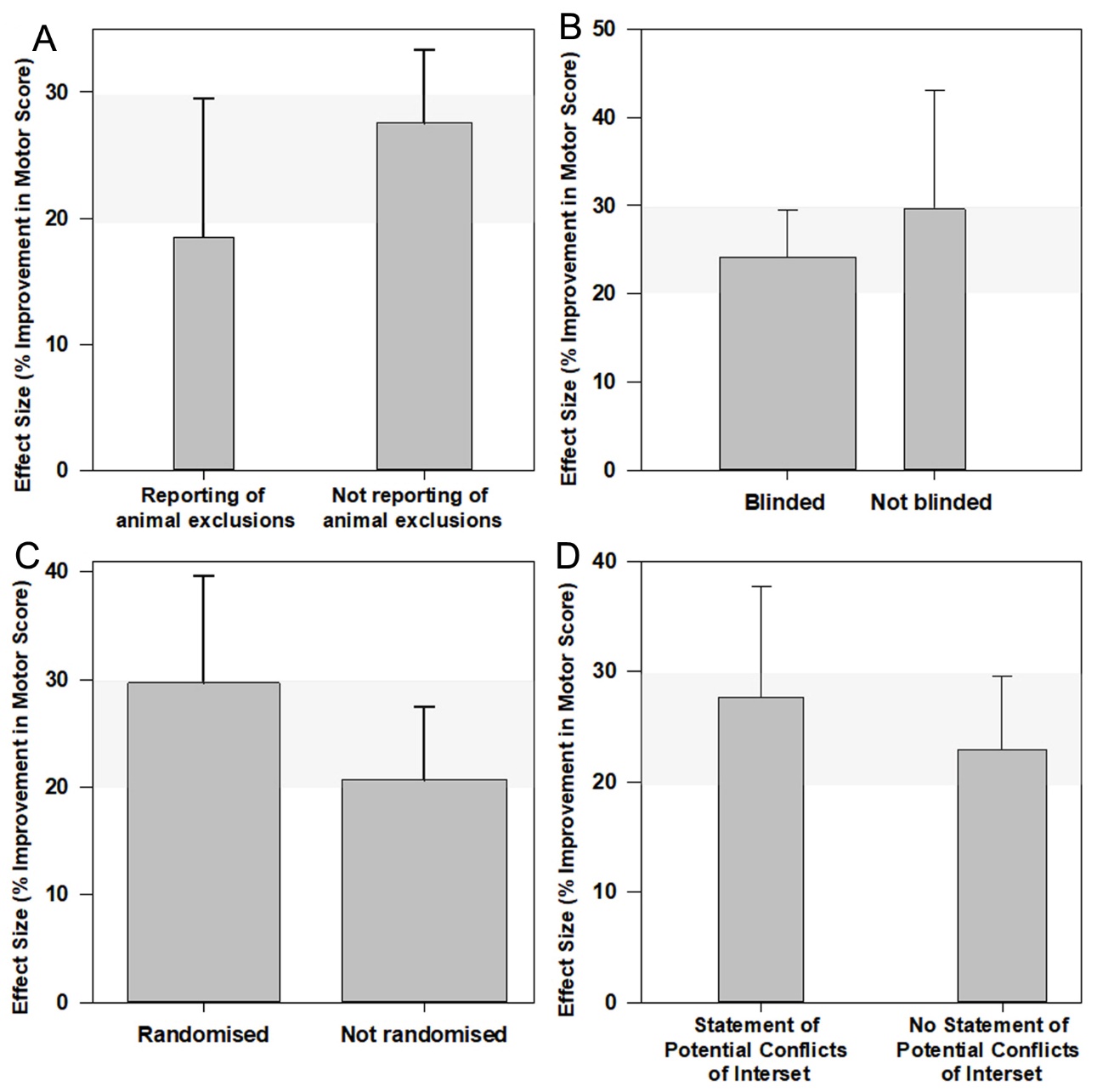


**eFigure 5:** **Differential effects of study quality on locomotor outcomes.**

We investigated the influence of reporting study quality variables on locomotor recovery. Effect of reporting, animal exclusions (A), blinding (B), randomisation (C) and conflicts of interest (D) on the percentage of improvement in motor score. Vertical error bars represent the 95% CI, and the horizontal shaded grey bar represents the 95% CI of all analysed studies. The width of each vertical bar is normalised to the square root of number of studies contributing to that comparison. Number of total comparisons: 102.


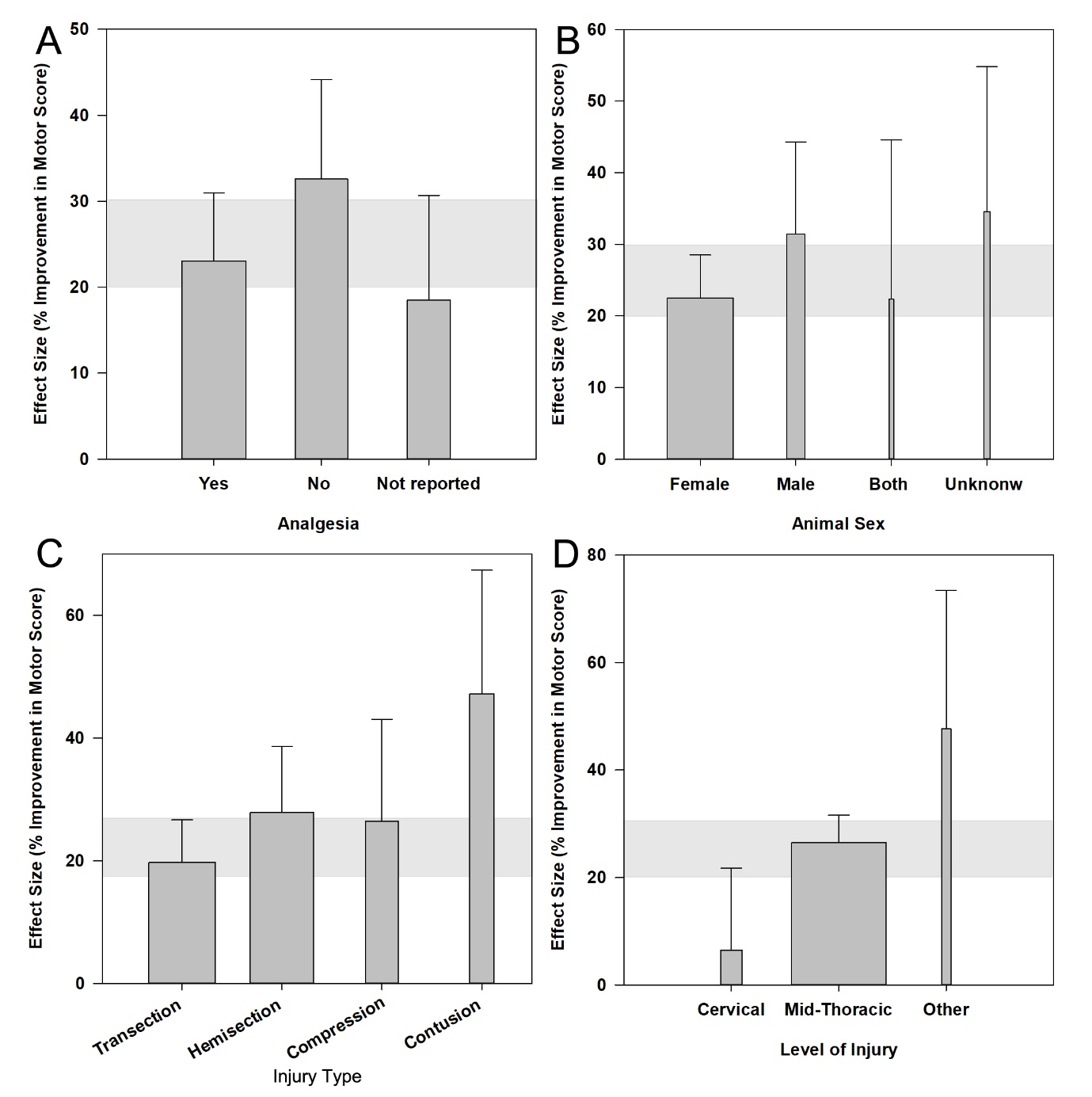


**eFigure 6:** **Differential effects of study design on locomotor outcomes**.

Meta-regression analysis of the effect of study design characteristics on the improvement in locomotor outcome. Effect of analgesic given (A), animal sex (B), injury type (C) and injury level (D) on the effect size as a percentage of improvement in motor score. Vertical error bars represent the 95% CI, and the horizontal shaded grey bar represents the 95% CI of all analysed studies. The width of each vertical bar is normalised to the square root of number of studies contributing to that comparison. Number of total comparisons: 102.


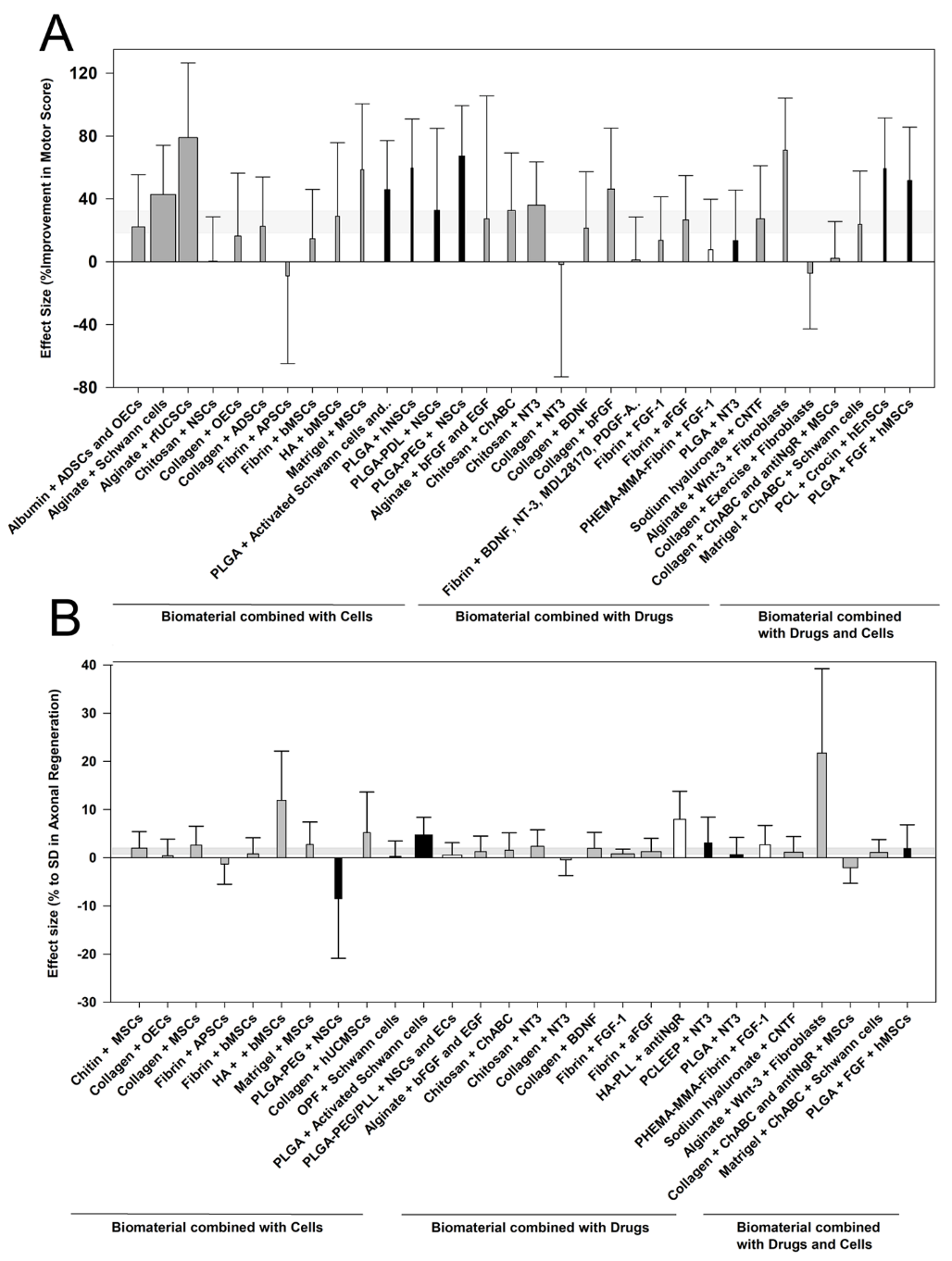


**eFigure 7:** **Different effect sizes of biomaterials-based combinations on locomotor and axonal regeneration outcomes**.

(A) Effect of the type of biomaterial-based combinations on the effect size as a percentage of improvement in motor score on studies performing only *in vivo* experiments. (B) Effect of the type of biomaterial-based combination on the effect size as improvement in axonal regeneration (SD) in studies performing only *in vivo* experiments. Variables are presented in alphabetical order and colour coded according to the biomaterial type: white for natural and synthetic, grey for natural and black for synthetic. Vertical error bars represent the 95% CI for the individual estimates, and the horizontal shaded grey bar represents the 95% CI of the global estimate. The width of each vertical bar is normalised to the square root of number of animals contributing to that comparison. Number of comparisons for locomotor recovery (A): 47. Number of comparisons for axonal regeneration (B): 103.


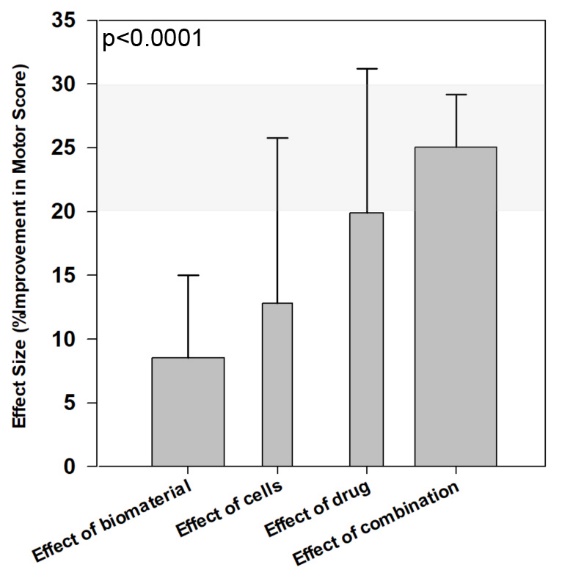


**eFigure 8: Influence of the treatment strategy on locomotor recovery outcomes.** Effect of the combination type (drugs, cells, or both) on the effect size as a percentage of improvement in motor score (n = 198). Vertical error bars represent the 95% CI for the individual estimates, and the horizontal shaded grey bar represents the 95% CI of the global estimate. The width of each vertical bar is normalised to the square root of number of animals contributing to that comparison.

**Online-only results for Objective3:**

Meta-regression analysis with grouped comparisons (Table 3; eFigure 7) did not find significant effects of combination strategies on LR (p=0.342, Tau^2^=438.4 and I^2^=92.8%, n=47) or *in vivo* AR (p=0.398, Tau^2^=2.7, I^2^ =77.3%, n=46). PLGA-based combinations were found to be associated with a large improvement of 38.0% (95% CI 16-60%) for LR and 1.7SD (95% CI 0.2-5.9SD) for *in vivo* AR.

**Online-only discussion**

**Neurotrophins**

Neurotrophic factors are a heterogeneous group of molecules involved in the development of the CNS and they promote robust neuronal survival and neurite outgrowth in the developing and adult CNS ([Lacroix and Tuszynski 2000](#_ENREF_63)). Early phase clinical trials have tested the efficacy of neurotrophins using gene therapy in patients with neurodegenerative diseases and SCI ([Tuszynski 2002](#_ENREF_119), [Silva, Sousa et al. 2014](#_ENREF_107)). One limitation of neurotrophins is that they selectively stimulate the outgrowth of subpopulation of neurons; for example BDNF promotes axonal regrowth of sensory but not corticospinal neurons ([Lacroix and Tuszynski 2000](#_ENREF_63)). Therefore, multiple trophic factors should be combined for a spinal cord repair therapy and their types and doses should be chosen and optimised carefully ([Lacroix and Tuszynski 2000](#_ENREF_63)).

**Angiogenic factors**

Recently angiogenesis has been appreciated as a key component to any CNS regenerative strategy because without new blood vessel formation waste products cannot be removed from the injury site and nutrients cannot be provided ([Sakiyama-Elbert, Johnson et al. 2012](#_ENREF_101)). The findings of this meta-analysis described all the angiogenic factors proposed to promote vascularisation after SCI. Their use in combination with biomaterials can help limit undesirable side effects, such as tumour growth and arthritis, potentially caused by their systemic and/or repeated delivery ([Haggerty, Maldonado-Lasuncion et al. 2018](#_ENREF_43)). Some studies have shown the beneficial effects of acute treatment of HA-PLGA + VEGF + BDNF in locomotor recovery and angiogenesis after SCI ([Wen, Yu et al. 2016](#_ENREF_129)) but the challenge now is to perform this chronically, perhaps in combination with endothelial cells ([Haggerty, Maldonado-Lasuncion et al. 2018](#_ENREF_43)).

**Cell therapy**

Cell therapy is an attractive therapeutic approach for SCI as it can provide significant neuroprotection, recovery through cell replacement, trophic support, and immune modulation ([Badner, Siddiqui et al. 2017](#_ENREF_5)). Despite these advantages there are still several challenges such as choice of cell type, cell harvesting and cell differentiation that impede translation of this therapy to the clinic ([Iyer, Wilems et al. 2017](#_ENREF_55)). Studies have suggested that NSCs and MSCs exert a clear therapeutic benefit. NSCs can differentiate into neurons or glial cells but autologous transplantation of them is not readily feasible ([Raspa, Pugliese et al. 2016](#_ENREF_94)). MSCs are a more appealing choice because of their ease for autologous transplantation and efficient expansion, yet their utility is confined to immunomodulatory and trophic effects and their neuronal differentiation is questioned ([Iyer, Wilems et al. 2017](#_ENREF_55)). Hence, fundamental questions regarding cell treatments still need to be clarified for integration into a combinatorial system to promote spinal cord repair.

**Abbreviation list**

| **Biomaterials**  Collagen-HSPG | Collagen-Heparan sulfate proteoglycan |
| --- | --- |
| ECM | Extracellular matrix |
| GG-GRGDS | Gellum gum-RGD |
| HA | Hyaluronic acid |
| HAMC | hyaluronic acid methylcellulose |
| HA-PLL | hyaluronan- poly(L-lysine) |
| HEMA-MOETACL | Hydroxyl ethyl methacrylate [2-(methacryloyloxy)ethyl]  trimethylammonium chloride |
| HPMA-RGD | N-(2-hydroxypropyl)methacrylamide-RGD |
| ION-Agarose | Iron Oxide Agarose Nanoparticles |
| LOCS | Linear ordered collagen scaffold |
| MAX8-Fmoc | MAX8-Fluorenylmethoxycarbonyl |
| OEGMA | Poly(ethylene glycol) methyl ether methacrylate |
| OPF | Oligo[(polyethylene glycol) fumarate] |
| PCL | Polyethylene glycol dimethacrylate |
| PCLEEP | Copolymer of Caprolactone and Ethyl Ethylene Phosphate |
| PEG | Polyethylene glycol |
| PEGDM | Poly (ethylene glycol) dimethacrylate |
| PF-127 | Pluronic F-12 |
| PHB-b-DEG | Polyhydroxybutyrate-b-polyethylene glycol 106 |
| PHB-HV | Poly (3-hydroxybutyrateco-3-hydroxyvalerate) |
| PHEMA-MMA | Poly(2-hydroxyethyl methacrylate-comethylmethacrylate) |
| PHPMA | 2-hydroxypropyl methacrylamide |
| PLGA | Poly(lactic-co-glycolic-acid) |
| PLGA-PDL | Poly(lactic-co-glycolic-acid) - Poly-D-lysine |
| PLLA | poly L-lactic acid |
| SAPs | self-assembling peptides |
| SWCNT | single-walled carbon nanotubes |
| **Drugs** |  |
| antiNgR | Nogo-66 Receptor Antibody |
| BDNF | Brain-Derived Neurotrophic Factor |
| aFGF | Acidic Fibroblast Growth Factor |
| bFGF | Basic Fibroblast Growth Factor |
| BMHP1 | Bone Marrow Homing Peptide 1 |
| ChABC | chondroitinase ABC |
| CNTF | Ciliary Neurotrophic Factor |
| dbcAMP | N(6),2'-O-dibutyryladenosine 3':5' cyclic monophosphate |
| ECM | Extra Cellular Matrix |
| EGF | Epidermal Growth Factor |
| EGFR | Epidermal Growth Factor Receptor |
| FAQ | FAQRVPP |
| FGF | Fibroblast Growth Factor |
| GDNF | Glial Cell-Derived Neurotrophic Factor |
| IGF-1 | Insulin-like growth factor 1 |
| IKVAV | Ile-Lys-Val-Ala-Val |
| Lingo-1 | Leucine-rich Repeat and Immunoglobulin Domain-containing  Nogo Receptor-Interacting Protein-1 |
| MDL28170 | Calpain Inhibitor III |
| NEP1-40 | Nogo-66 receptor antagonist peptide |
| NGF | Nerve Growth Factor |
| NT3 | Neurotrophin-3 |
| PDGF-A | Platelet-Derived Growth Factor subunit A |
| RGD | Arginylglycylaspartic acid |
| SB216763 | Glycogen Synthase Kinase-3 Inhibitor |
| SDF | Stromal Cell-Derived Factor |
| VEGF | Vascular Endothelial Growth Factor |
| Wnt-3 | Wnt family member 3 |
| **Cells** |  |
| ADSCs | Adipose-Derived Stem Cells |
| APSCs | Adipose progenitor Stem Cells |
| bMSCs | Bone marrow stromal cells |
| DPSCs | Dental Pulp Stem Cells |
| ECs | Endothelial Cells |
| hADSCs | human Adipose-Derived Stem Cells |
| hEnSCs | human Endometrial Stem Cells |
| hfNSCs | human fetal Neuro Stem Cells |
| hMSCs | human Mesenchymal Stem Cells |
| hNSCs | human Neuro Stem Cells |
| hOMSCs | human Oral Mucosa Stem Cells |
| hUCMSCs | human Umbilical Cord Blood Derived-Mesenchymal Stem Cells |
| MSCs | Mesenchymal Stem Cells |
| NSCs | Neuro Stem Cells |
| NSPCs | Neuro Stem/Progenitor Cells |
| OECs | Olfactory Ensheathing cells |
| OPCs | Oligodendrocyte Progenitor Cells |
| rfUCSCs | rat fetal Umbilical Cord Stem cells |
| SCs | Schwann cells |

**References**

Altinova, H., S. Mollers, R. Deumens, J. Gerardo-Nava, T. Fuhrmann, S. G. A. van Neerven, A. Bozkurt, C. A. Mueller, H. J. Hoff, I. Heschel, J. Weis and G. A. Brook (2016). "Functional recovery not correlated with axon regeneration through olfactory ensheathing cell-seeded scaffolds in a model of acute spinal cord injury." Tissue Eng Regen Med **13**(5): 585-600.

Anderson, M. A., J. E. Burda, Y. Ren, Y. Ao, T. M. O'Shea, R. Kawaguchi, G. Coppola, B. S. Khakh, T. J. Deming and M. V. Sofroniew (2016). "Astrocyte scar formation aids central nervous system axon regeneration." Nature **532**(7598): 195-200.

Ansorena, E., P. De Berdt, B. Ucakar, T. Simon-Yarza, D. Jacobs, O. Schakman, A. Jankovski, R. Deumens, M. J. Blanco-Prieto, V. Preat and A. des Rieux (2013). "Injectable alginate hydrogel loaded with GDNF promotes functional recovery in a hemisection model of spinal cord injury." Int J Pharm **455**(1-2): 148-158.

Asmani, M. N., J. Ai, G. Amoabediny, A. Noroozi, M. Azami, S. Ebrahimi-Barough, M. Navaei-Nigjeh, A. Ai and M. Jafarabadi (2013). "Three-dimensional culture of differentiated endometrial stromal cells to oligodendrocyte progenitor cells (OPCs) in fibrin hydrogel." Cell biology international **37**(12): 1340-1349.

Badner, A., A. M. Siddiqui and M. G. Fehlings (2017). "Spinal cord injuries: how could cell therapy help?" Expert Opinion on Biological Therapy **17**(5): 529-541.

Baumann, M. D., C. E. Kang, J. C. Stanwick, Y. Wang, H. Kim, Y. Lapitsky and M. S. Shoichet (2009). "An injectable drug delivery platform for sustained combination therapy." Journal of controlled release : official journal of the Controlled Release Society **138**(3): 205-213.

Baumann, M. D., C. E. Kang, C. H. Tator and M. S. Shoichet (2010). "Intrathecal delivery of a polymeric nanocomposite hydrogel after spinal cord injury." Biomaterials **31**(30): 7631-7639.

Berns, E. J., Z. Alvarez, J. E. Goldberger, J. Boekhoven, J. A. Kessler, H. G. Kuhn and S. I. Stupp (2016). "A tenascin-C mimetic peptide amphiphile nanofiber gel promotes neurite outgrowth and cell migration of neurosphere-derived cells." Acta biomaterialia **37**: 50-58.

Bozkurt, G., A. J. Mothe, T. Zahir, H. Kim, M. S. Shoichet and C. H. Tator (2010). "Chitosan channels containing spinal cord-derived stem/progenitor cells for repair of subacute spinal cord injury in the rat." Neurosurgery **67**(6): 1733-1744.

Breen, B. A., H. Kraskiewicz, R. Ronan, A. Kshiragar, A. Patar, T. Sargeant, A. Pandit and S. S. McMahon (2017). "Therapeutic Effect of Neurotrophin-3 Treatment in an Injectable Collagen Scaffold Following Rat Spinal Cord Hemisection Injury." Acs Biomaterials Science & Engineering **3**(7): 1287-1295.

Burdick, J. A., M. Ward, E. Liang, M. J. Young and R. Langer (2006). "Stimulation of neurite outgrowth by neurotrophins delivered from degradable hydrogels." Biomaterials **27**(3): 452-459.

Buzoianu-Anguiano, V., S. Orozco-Suarez, E. Garcia-Vences, S. Caballero-Chacon, G. Guizar-Sahagun, L. Chavez-Sanchez and I. Grijalva (2015). "The Morphofunctional Effect of the Transplantation of Bone Marrow Stromal Cells and Predegenerated Peripheral Nerve in Chronic Paraplegic Rat Model via Spinal Cord Transection." Neural plasticity.

Caron, I., F. Rossi, S. Papa, R. Aloe, M. Sculco, E. Mauri, A. Sacchetti, E. Erba, N. Panini, V. Parazzi, M. Barilani, G. Forloni, G. Perale, L. Lazzari and P. Veglianese (2016). "A new three dimensional biomimetic hydrogel to deliver factors secreted by human mesenchymal stem cells in spinal cord injury." Biomaterials **75**: 135-147.

Chan, R. T., R. A. Russell, H. Marcal, T. H. Lee, P. J. Holden and L. J. Foster (2014). "BioPEGylation of polyhydroxybutyrate promotes nerve cell health and migration." Biomacromolecules **15**(1): 339-349.

Chen, B., J. He, H. Yang, Q. Zhang, L. Zhang, X. Zhang, E. Xie, C. Liu, R. Zhang, Y. Wang, L. Huang and D. Hao (2015). "Repair of spinal cord injury by implantation of bFGF-incorporated HEMA-MOETACL hydrogel in rats." Sci Rep **5**: 9017.

Chen, J., Z. Zhang, J. Liu, R. Zhou, X. Zheng, T. Chen, L. Wang, M. Huang, C. Yang, Z. Li, C. Yang, X. Bai and D. Jin (2014). "Acellular spinal cord scaffold seeded with bone marrow stromal cells protects tissue and promotes functional recovery in spinal cord-injured rats." J Neurosci Res **92**(3): 307-317.

Chen, X., Y. Yang, J. Yao, W. Lin, Y. Li, Y. Chen, Y. Gao, Y. Yang, X. Gu and X. Wang (2011). "Bone marrow stromal cells-loaded chitosan conduits promote repair of complete transection injury in rat spinal cord." J Mater Sci Mater Med **22**(10): 2347-2356.

Cholas, R., H. P. Hsu and M. Spector (2012). "Collagen scaffolds incorporating select therapeutic agents to facilitate a reparative response in a standardized hemiresection defect in the rat spinal cord." Tissue Eng Part A **18**(19-20): 2158-2172.

Cholas, R. H., H. P. Hsu and M. Spector (2012). "The reparative response to cross-linked collagen-based scaffolds in a rat spinal cord gap model." Biomaterials **33**(7): 2050-2059.

Cigognini, D., A. Satta, B. Colleoni, D. Silva, M. Donega, S. Antonini and F. Gelain (2011). "Evaluation of early and late effects into the acute spinal cord injury of an injectable functionalized self-assembling scaffold." PLoS One **6**(5): e19782.

De Berdt, P., J. Vanacker, B. Ucakar, L. Elens, A. Diogenes, J. G. Leprince, R. Deumens and A. des Rieux (2015). "Dental Apical Papilla as Therapy for Spinal Cord Injury." J Dent Res **94**(11): 1575-1581.

DePaul, M. A., C. Y. Lin, J. Silver and Y. S. Lee (2015). "Peripheral Nerve Transplantation Combined with Acidic Fibroblast Growth Factor and Chondroitinase Induces Regeneration and Improves Urinary Function in Complete Spinal Cord Transected Adult Mice." PLoS One **10**(10): e0139335.

des Rieux, A., P. De Berdt, E. Ansorena, B. Ucakar, J. Damien, O. Schakman, E. Audouard, C. Bouzin, D. Auhl, T. Simon-Yarza, O. Feron, M. J. Blanco-Prieto, P. Carmeliet, C. Bailly, F. Clotman and V. Preat (2014). "Vascular endothelial growth factor-loaded injectable hydrogel enhances plasticity in the injured spinal cord." J Biomed Mater Res A **102**(7): 2345-2355.

Deumens, R., S. F. Van Gorp, A. Bozkurt, C. Beckmann, T. Fuhrmann, K. Montzka, R. Tolba, E. Kobayashi, I. Heschel, J. Weis and G. A. Brook (2013). "Motor outcome and allodynia are largely unaffected by novel olfactory ensheathing cell grafts to repair low-thoracic lesion gaps in the adult rat spinal cord." Behav Brain Res **237**: 185-189.

Downing, T. L., A. Wang, Z. Q. Yan, Y. Nout, A. L. Lee, M. S. Beattie, J. C. Bresnahan, D. L. Farmer and S. Li (2012). "Drug-eluting microfibrous patches for the local delivery of rolipram in spinal cord repair." J Control Release **161**(3): 910-917.

Elias, P. Z., G. W. Liu, H. Wei, M. C. Jensen, P. J. Horner and S. H. Pun (2015). "A functionalized, injectable hydrogel for localized drug delivery with tunable thermosensitivity: synthesis and characterization of physical and toxicological properties." J Control Release **208**: 76-84.

Elliott Donaghue, I., C. H. Tator and M. S. Shoichet (2016). "Local Delivery of Neurotrophin-3 and Anti-NogoA Promotes Repair After Spinal Cord Injury." Tissue Eng Part A **22**(9-10): 733-741.

Erdogan, B., M. Bavbek, I. F. Sahin, H. Caner, O. Ozen, E. B. Denkbas and M. N. Altinors (2010). "Fetal allogeneic umbilical cord cell transplantation improves motor function in spinal cord-injured rats." Turk Neurosurg **20**(3): 286-294.

Fan, C., X. Li, Z. Xiao, Y. Zhao, H. Liang, B. Wang, S. Han, B. Xu, N. Wang, S. Liu, W. Xue and J. Dai (2017). "A modified collagen scaffold facilitates endogenous neurogenesis for acute spinal cord injury repair." Acta Biomater **51**: 304-316.

Fan, J., Z. Xiao, H. Zhang, B. Chen, G. Tang, X. Hou, W. Ding, B. Wang, P. Zhang, J. Dai and R. Xu (2010). "Linear ordered collagen scaffolds loaded with collagen-binding neurotrophin-3 promote axonal regeneration and partial functional recovery after complete spinal cord transection." J Neurotrauma **27**(9): 1671-1683.

Fan, J., H. Zhang, J. He, Z. Xiao, B. Chen, J. Xiaodan, J. Dai and R. Xu (2011). "Neural regrowth induced by PLGA nerve conduits and neurotrophin-3 in rats with complete spinal cord transection." J Biomed Mater Res B Appl Biomater **97**(2): 271-277.

Ferrero-Gutierrez, A., Y. Menendez-Menendez, M. Alvarez-Viejo, A. Meana and J. Otero (2013). "New serum-derived albumin scaffold seeded with adipose-derived stem cells and olfactory ensheathing cells used to treat spinal cord injured rats." Histol Histopathol **28**(1): 89-100.

Fouad, K., L. Schnell, M. B. Bunge, M. E. Schwab, T. Liebscher and D. D. Pearse (2005). "Combining Schwann cell bridges and olfactory-ensheathing glia grafts with chondroitinase promotes locomotor recovery after complete transection of the spinal cord." J Neurosci **25**(5): 1169-1178.

Francis, N. L., P. M. Hunger, A. E. Donius, U. G. Wegst and M. A. Wheatley (2017). "Strategies for neurotrophin-3 and chondroitinase ABC release from freeze-cast chitosan-alginate nerve-guidance scaffolds." J Tissue Eng Regen Med **11**(1): 285-294.

Fuhrmann, T., J. Obermeyer, C. H. Tator and M. S. Shoichet (2015). "Click-crosslinked injectable hyaluronic acid hydrogel is safe and biocompatible in the intrathecal space for ultimate use in regenerative strategies of the injured spinal cord." Methods **84**: 60-69.

Fuhrmann, T., R. Y. Tam, B. Ballarin, B. Coles, I. Elliott Donaghue, D. van der Kooy, A. Nagy, C. H. Tator, C. M. Morshead and M. S. Shoichet (2016). "Injectable hydrogel promotes early survival of induced pluripotent stem cell-derived oligodendrocytes and attenuates longterm teratoma formation in a spinal cord injury model." Biomaterials **83**: 23-36.

Ganz, J., E. Shor, S. Guo, A. Sheinin, I. Arie, I. Michaelevski, S. Pitaru, D. Offen and S. Levenberg (2017). "Implantation of 3D Constructs Embedded with Oral Mucosa-Derived Cells Induces Functional Recovery in Rats with Complete Spinal Cord Transection." Front Neurosci **11**: 589.

Gelain, F., D. Cigognini, A. Caprini, D. Silva, B. Colleoni, M. Donega, S. Antonini, B. E. Cohen and A. Vescovi (2012). "New bioactive motifs and their use in functionalized self-assembling peptides for NSC differentiation and neural tissue engineering." Nanoscale **4**(9): 2946-2957.

Gomes, E. D., S. S. Mendes, H. Leite-Almeida, J. M. Gimble, R. Y. Tam, M. S. Shoichet, N. Sousa, N. A. Silva and A. J. Salgado (2016). "Combination of a peptide-modified gellan gum hydrogel with cell therapy in a lumbar spinal cord injury animal model." Biomaterials **105**: 38-51.

Grulova, I., L. Slovinska, J. Blasko, S. Devaux, M. Wisztorski, M. Salzet, I. Fournier, O. Kryukov, S. Cohen and D. Cizkova (2015). "Delivery of Alginate Scaffold Releasing Two Trophic Factors for Spinal Cord Injury Repair." Sci Rep **5**: 13702.

Gunther, M. I., N. Weidner, R. Muller and A. Blesch (2015). "Cell-seeded alginate hydrogel scaffolds promote directed linear axonal regeneration in the injured rat spinal cord." Acta Biomater **27**: 140-150.

Gupta, D., C. H. Tator and M. S. Shoichet (2006). "Fast-gelling injectable blend of hyaluronan and methylcellulose for intrathecal, localized delivery to the injured spinal cord." Biomaterials **27**(11): 2370-2379.

Haggerty, A. E., I. Maldonado-Lasuncion and M. Oudega (2018). "Biomaterials for revascularization and immunomodulation after spinal cord injury." Biomedical Materials **13**(4): 14.

Hakim, J. S., M. Esmaeili Rad, P. J. Grahn, B. K. Chen, A. M. Knight, A. M. Schmeichel, N. A. Isaq, M. Dadsetan, M. J. Yaszemski and A. J. Windebank (2015). "Positively Charged Oligo[Poly(Ethylene Glycol) Fumarate] Scaffold Implantation Results in a Permissive Lesion Environment after Spinal Cord Injury in Rat." Tissue Eng Part A **21**(13-14): 2099-2114.

Han, Q., W. Jin, Z. Xiao, H. Ni, J. Wang, J. Kong, J. Wu, W. Liang, L. Chen, Y. Zhao, B. Chen and J. Dai (2010). "The promotion of neural regeneration in an extreme rat spinal cord injury model using a collagen scaffold containing a collagen binding neuroprotective protein and an EGFR neutralizing antibody." Biomaterials **31**(35): 9212-9220.

Han, Q., W. Sun, H. Lin, W. Zhao, Y. Gao, Y. Zhao, B. Chen, Z. Xiao, W. Hu, Y. Li, B. Yang and J. Dai (2009). "Linear ordered collagen scaffolds loaded with collagen-binding brain-derived neurotrophic factor improve the recovery of spinal cord injury in rats." Tissue Eng Part A **15**(10): 2927-2935.

Han, S., B. Wang, W. Jin, Z. Xiao, B. Chen, H. Xiao, W. Ding, J. Cao, F. Ma, X. Li, B. Yuan, T. Zhu, X. Hou, J. Wang, J. Kong, W. Liang and J. Dai (2014). "The collagen scaffold with collagen binding BDNF enhances functional recovery by facilitating peripheral nerve infiltrating and ingrowth in canine complete spinal cord transection." Spinal Cord **52**(12): 867-873.

Han, S., B. Wang, W. Jin, Z. Xiao, X. Li, W. Ding, M. Kapur, B. Chen, B. Yuan, T. Zhu, H. Wang, J. Wang, Q. Dong, W. Liang and J. Dai (2015). "The linear-ordered collagen scaffold-BDNF complex significantly promotes functional recovery after completely transected spinal cord injury in canine." Biomaterials **41**: 89-96.

He, Q., T. Zhang, Y. Yang and F. Ding (2009). "In vitro biocompatibility of chitosan-based materials to primary culture of hippocampal neurons." J Mater Sci Mater Med **20**(7): 1457-1466.

Hejcl, A., J. Sedy, M. Kapcalova, D. A. Toro, T. Amemori, P. Lesny, K. Likavcanova-Masinova, E. Krumbholcova, M. Pradny, J. Michalek, M. Burian, M. Hajek, P. Jendelova and E. Sykova (2010). "HPMA-RGD hydrogels seeded with mesenchymal stem cells improve functional outcome in chronic spinal cord injury." Stem Cells Dev **19**(10): 1535-1546.

Hou, T., Y. Wu, L. Wang, Y. Liu, L. Zeng, M. Li, Z. Long, H. Chen, Y. Li and Z. Wang (2012). "Cellular prostheses fabricated with motor neurons seeded in self-assembling peptide promotes partial functional recovery after spinal cord injury in rats." Tissue Eng Part A **18**(9-10): 974-985.

Hsueh, Y. Y., Y. L. Chiang, C. C. Wu and S. C. Lin (2012). "Spheroid formation and neural induction in human adipose-derived stem cells on a chitosan-coated surface." Cells Tissues Organs **196**(2): 117-128.

Hwang, D. H., H. M. Kim, Y. M. Kang, I. S. Joo, C. S. Cho, B. W. Yoon, S. U. Kim and B. G. Kim (2011). "Combination of multifaceted strategies to maximize the therapeutic benefits of neural stem cell transplantation for spinal cord repair." Cell Transplant **20**(9): 1361-1379.

Itosaka, H., S. Kuroda, H. Shichinohe, H. Yasuda, S. Yano, S. Kamei, R. Kawamura, K. Hida and Y. Iwasaki (2009). "Fibrin matrix provides a suitable scaffold for bone marrow stromal cells transplanted into injured spinal cord: a novel material for CNS tissue engineering." Neuropathology **29**(3): 248-257.

Iyer, N. R., T. S. Wilems and S. E. Sakiyama-Elbert (2017). "Stem cells for spinal cord injury: Strategies to inform differentiation and transplantation." Biotechnology and Bioengineering **114**(2): 245-259.

Jian, R., Y. Yixu, L. Sheyu, S. Jianhong, Y. Yaohua, S. Xing, H. Qingfeng, L. Xiaojian, Z. Lei, Z. Yan, X. Fangling, G. Huasong and G. Yilu (2015). "Repair of spinal cord injury by chitosan scaffold with glioma ECM and SB216763 implantation in adult rats." J Biomed Mater Res A **103**(10): 3259-3272.

Jiao, G., G. Lou, Y. Mo, Y. Pan, Z. Zhang, R. Guo and Z. Li (2017). "A combination of GDNF and hUCMSC transplantation loaded on SF/AGs composite scaffolds for spinal cord injury repair." Mater Sci Eng C Mater Biol Appl **74**: 230-237.

Johnson, P. J., S. R. Parker and S. E. Sakiyama-Elbert (2009). "Controlled release of neurotrophin-3 from fibrin-based tissue engineering scaffolds enhances neural fiber sprouting following subacute spinal cord injury." Biotechnol Bioeng **104**(6): 1207-1214.

Kabiri, M., S. Oraee-Yazdani, M. Dodel, H. Hanaee-Ahvaz, S. Soudi, E. Seyedjafari, M. Salehi and M. Soleimani (2015). "Cytocompatibility of a conductive nanofibrous carbon nanotube/poly (L-Lactic acid) composite scaffold intended for nerve tissue engineering." EXCLI J **14**: 851-860.

Kang, C. E., M. D. Baumann, C. H. Tator and M. S. Shoichet (2013). "Localized and sustained delivery of fibroblast growth factor-2 from a nanoparticle-hydrogel composite for treatment of spinal cord injury." Cells Tissues Organs **197**(1): 55-63.

Kim, H., T. Zahir, C. H. Tator and M. S. Shoichet (2011). "Effects of dibutyryl cyclic-AMP on survival and neuronal differentiation of neural stem/progenitor cells transplanted into spinal cord injured rats." PLoS One **6**(6): e21744.

Kueh, J. L., D. Li, G. Raisman, D. Jenkins, Y. Li and R. Stevens (2012). "Directionality and bipolarity of olfactory ensheathing cells on electrospun nanofibers." Nanomedicine (Lond) **7**(8): 1211-1224.

Lacroix, S. and M. H. Tuszynski (2000). "Neurotrophic factors and gene therapy in spinal cord injury." Neurorehabil Neural Repair **14**(4): 265-275.

Landis, S. C., S. G. Amara, K. Asadullah, C. P. Austin, R. Blumenstein, E. W. Bradley, R. G. Crystal, R. B. Darnell, R. J. Ferrante, H. Fillit, R. Finkelstein, M. Fisher, H. E. Gendelman, R. M. Golub, J. L. Goudreau, R. A. Gross, A. K. Gubitz, S. E. Hesterlee, D. W. Howells, J. Huguenard, K. Kelner, W. Koroshetz, D. Krainc, S. E. Lazic, M. S. Levine, M. R. Macleod, J. M. McCall, R. T. Moxley, 3rd, K. Narasimhan, L. J. Noble, S. Perrin, J. D. Porter, O. Steward, E. Unger, U. Utz and S. D. Silberberg (2012). "A call for transparent reporting to optimize the predictive value of preclinical research." Nature **490**(7419): 187-191.

Lee, Y. S., I. Hsiao and V. W. Lin (2002). "Peripheral nerve grafts and aFGF restore partial hindlimb function in adult paraplegic rats." J Neurotrauma **19**(10): 1203-1216.

Lee, Y. S., C. Y. Lin, V. J. Caiozzo, R. T. Robertson, J. Yu and V. W. Lin (2007). "Repair of spinal cord transection and its effects on muscle mass and myosin heavy chain isoform phenotype." J Appl Physiol (1985) **103**(5): 1808-1814.

Li, G., M. T. Che, K. Zhang, L. N. Qin, Y. T. Zhang, R. Q. Chen, L. M. Rong, S. Liu, Y. Ding, H. Y. Shen, S. M. Long, J. L. Wu, E. A. Ling and Y. S. Zeng (2016). "Graft of the NT-3 persistent delivery gelatin sponge scaffold promotes axon regeneration, attenuates inflammation, and induces cell migration in rat and canine with spinal cord injury." Biomaterials **83**: 233-248.

Li, X., J. Han, Y. Zhao, W. Ding, J. Wei, J. Li, S. Han, X. Shang, B. Wang, B. Chen, Z. Xiao and J. Dai (2016). "Functionalized collagen scaffold implantation and cAMP administration collectively facilitate spinal cord regeneration." Acta Biomater **30**: 233-245.

Li, X., Z. Xiao, J. Han, L. Chen, H. Xiao, F. Ma, X. Hou, X. Li, J. Sun, W. Ding, Y. Zhao, B. Chen and J. Dai (2013). "Promotion of neuronal differentiation of neural progenitor cells by using EGFR antibody functionalized collagen scaffolds for spinal cord injury repair." Biomaterials **34**(21): 5107-5116.

Li, X., Y. Zhao, S. Cheng, S. Han, M. Shu, B. Chen, X. Chen, F. Tang, N. Wang, Y. Tu, B. Wang, Z. Xiao, S. Zhang and J. Dai (2017). "Cetuximab modified collagen scaffold directs neurogenesis of injury-activated endogenous neural stem cells for acute spinal cord injury repair." Biomaterials **137**: 73-86.

Lindsey, S., J. H. Piatt, P. Worthington, C. Sonmez, S. Satheye, J. P. Schneider, D. J. Pochan and S. A. Langhans (2015). "Beta Hairpin Peptide Hydrogels as an Injectable Solid Vehicle for Neurotrophic Growth Factor Delivery." Biomacromolecules **16**(9): 2672-2683.

Liu, C., Y. Huang, M. Pang, Y. Yang, S. Li, L. Liu, T. Shu, W. Zhou, X. Wang, L. Rong and B. Liu (2015). "Tissue-engineered regeneration of completely transected spinal cord using induced neural stem cells and gelatin-electrospun poly (lactide-co-glycolide)/polyethylene glycol scaffolds." PLoS One **10**(3): e0117709.

Liu, J., Q. Chen, Z. Zhang, Y. Zheng, X. Sun, X. Cao, A. Gong, Y. Cui, Q. He and P. Jiang (2013). "Fibrin scaffolds containing ectomesenchymal stem cells enhance behavioral and histological improvement in a rat model of spinal cord injury." Cells Tissues Organs **198**(1): 35-46.

Lu, P., Y. Wang, L. Graham, K. McHale, M. Gao, D. Wu, J. Brock, A. Blesch, E. S. Rosenzweig, L. A. Havton, B. Zheng, J. M. Conner, M. Marsala and M. H. Tuszynski (2012). "Long-distance growth and connectivity of neural stem cells after severe spinal cord injury." Cell **150**(6): 1264-1273.

Macaya, D. J., K. Hayakawa, K. Arai and M. Spector (2013). "Astrocyte infiltration into injectable collagen-based hydrogels containing FGF-2 to treat spinal cord injury." Biomaterials **34**(14): 3591-3602.

Milbreta, U., L. H. Nguyen, H. Diao, J. Lin, W. Wu, C.-Y. Sun, J. Wang and S. Y. Chew (2016). "Three-Dimensional Nanofiber Hybrid Scaffold Directs and Enhances Axonal Regeneration after Spinal Cord Injury." ACS Biomaterials Science & Engineering **2**(8): 1319-1329.

Nguyen, V. T., S. C. Ko, G. W. Oh, S. Y. Heo, Y. J. Jeon, W. S. Park, I. W. Choi, S. W. Choi and W. K. Jung (2016). "Anti-inflammatory effects of sodium alginate/gelatine porous scaffolds merged with fucoidan in murine microglial BV2 cells." Int J Biol Macromol **93**(Pt B): 1620-1632.

Ni, S., T. Xia, X. Li, X. Zhu, H. Qi, S. Huang and J. Wang (2015). "Sustained delivery of chondroitinase ABC by poly(propylene carbonate)-chitosan micron fibers promotes axon regeneration and functional recovery after spinal cord hemisection." Brain Res **1624**: 469-478.

Nomura, H., B. Baladie, Y. Katayama, C. M. Morshead, M. S. Shoichet and C. H. Tator (2008). "Delayed implantation of intramedullary chitosan channels containing nerve grafts promotes extensive axonal regeneration after spinal cord injury." Neurosurgery **63**(1): 127-141; discussion 141-123.

Nomura, H., Y. Katayama, M. S. Shoichet and C. H. Tator (2006). "Complete spinal cord transection treated by implantation of a reinforced synthetic hydrogel channel results in syringomyelia and caudal migration of the rostral stump." Neurosurgery **59**(1): 183-192; discussion 183-192.

Nomura, H., T. Zahir, H. Kim, Y. Katayama, I. Kulbatski, C. M. Morshead, M. S. Shoichet and C. H. Tator (2008). "Extramedullary chitosan channels promote survival of transplanted neural stem and progenitor cells and create a tissue bridge after complete spinal cord transection." Tissue Eng Part A **14**(5): 649-665.

Nothias, J. M., T. Mitsui, J. S. Shumsky, I. Fischer, M. D. Antonacci and M. Murray (2005). "Combined effects of neurotrophin secreting transplants, exercise, and serotonergic drug challenge improve function in spinal rats." Neurorehabil Neural Repair **19**(4): 296-312.

Pakulska, M. M., C. H. Tator and M. S. Shoichet (2017). "Local delivery of chondroitinase ABC with or without stromal cell-derived factor 1alpha promotes functional repair in the injured rat spinal cord." Biomaterials **134**: 13-21.

Pal, A., A. Singh, T. C. Nag, P. Chattopadhyay, R. Mathur and S. Jain (2013). "Iron oxide nanoparticles and magnetic field exposure promote functional recovery by attenuating free radical-induced damage in rats with spinal cord transection." Int J Nanomedicine **8**: 2259-2272.

Park, J., E. Lim, S. Back, H. Na, Y. Park and K. Sun (2010). "Nerve regeneration following spinal cord injury using matrix metalloproteinase-sensitive, hyaluronic acid-based biomimetic hydrogel scaffold containing brain-derived neurotrophic factor." J Biomed Mater Res A **93**(3): 1091-1099.

Park, J. H., J. Min, S. R. Baek, S. W. Kim, I. K. Kwon and S. R. Jeon (2013). "Enhanced neuroregenerative effects by scaffold for the treatment of a rat spinal cord injury with Wnt3a-secreting fibroblasts." Acta Neurochir (Wien) **155**(5): 809-816.

Park, S. S., Y. J. Lee, S. H. Lee, D. Lee, K. Choi, W. H. Kim, O. K. Kweon and H. J. Han (2012). "Functional recovery after spinal cord injury in dogs treated with a combination of Matrigel and neural-induced adipose-derived mesenchymal Stem cells." Cytotherapy **14**(5): 584-597.

Patel, V., G. Joseph, A. Patel, S. Patel, D. Bustin, D. Mawson, L. M. Tuesta, R. Puentes, M. Ghosh and D. D. Pearse (2010). "Suspension matrices for improved Schwann-cell survival after implantation into the injured rat spinal cord." J Neurotrauma **27**(5): 789-801.

Peng, Z., W. Gao, B. Yue, J. Jiang, Y. Gu, J. Dai, L. Chen and Q. Shi (2016). "Promotion of neurological recovery in rat spinal cord injury by mesenchymal stem cells loaded on nerve-guided collagen scaffold through increasing alternatively activated macrophage polarization." J Tissue Eng Regen Med.

Pertici, V., J. Amendola, J. Laurin, D. Gigmes, L. Madaschi, S. Carelli, T. Marqueste, A. Gorio and P. Decherchi (2013). "The use of poly(N-[2-hydroxypropyl]-methacrylamide) hydrogel to repair a T10 spinal cord hemisection in rat: a behavioural, electrophysiological and anatomical examination." ASN Neuro **5**(2): 149-166.

Pertici, V., T. Trimaille, J. Laurin, M. S. Felix, T. Marqueste, B. Pettmann, J. P. Chauvin, D. Gigmes and P. Decherchi (2014). "Repair of the injured spinal cord by implantation of a synthetic degradable block copolymer in rat." Biomaterials **35**(24): 6248-6258.

Pritchard, C. D., J. R. Slotkin, D. Yu, H. Dai, M. S. Lawrence, R. T. Bronson, F. M. Reynolds, Y. D. Teng, E. J. Woodard and R. S. Langer (2010). "Establishing a model spinal cord injury in the African green monkey for the preclinical evaluation of biodegradable polymer scaffolds seeded with human neural stem cells." J Neurosci Methods **188**(2): 258-269.

Rad, I., H. Mobasheri, F. Najafi and M. Rezaei (2014). "Efficient repairing effect of PEG based tri-block copolymer on mechanically damaged PC12 cells and isolated spinal cord." J Mater Sci Mater Med **25**(6): 1539-1551.

Raspa, A., R. Pugliese, M. Maleki and F. Gelain (2016). "Recent Therapeutic Approaches for Spinal Cord Injury." Biotechnology and Bioengineering **113**(2): 253-259.

Rauch, M. F., S. R. Hynes, J. Bertram, A. Redmond, R. Robinson, C. Williams, H. Xu, J. A. Madri and E. B. Lavik (2009). "Engineering angiogenesis following spinal cord injury: a coculture of neural progenitor and endothelial cells in a degradable polymer implant leads to an increase in vessel density and formation of the blood-spinal cord barrier." Eur J Neurosci **29**(1): 132-145.

Raynald, Y. Li, H. Yu, H. Huang, M. Guo, R. Hua, F. Jiang, K. Zhang, H. Li, F. Wang, L. Li, F. Cui and Y. An (2016). "The hetero-transplantation of human bone marrow stromal cells carried by hydrogel unexpectedly demonstrates a significant role in the functional recovery in the injured spinal cord of rats." Brain Res **1634**: 21-33.

Ribeiro-Samy, S., N. A. Silva, V. M. Correlo, J. S. Fraga, L. Pinto, A. Teixeira-Castro, H. Leite-Almeida, A. Almeida, J. M. Gimble, N. Sousa, A. J. Salgado and R. L. Reis (2013). "Development and characterization of a PHB-HV-based 3D scaffold for a tissue engineering and cell-therapy combinatorial approach for spinal cord injury regeneration." Macromol Biosci **13**(11): 1576-1592.

Rooney, G. E., A. M. Knight, N. N. Madigan, L. Gross, B. Chen, C. V. Giraldo, S. Seo, J. J. Nesbitt, M. Dadsetan, M. J. Yaszemski and A. J. Windebank (2011). "Sustained delivery of dibutyryl cyclic adenosine monophosphate to the transected spinal cord via oligo [(polyethylene glycol) fumarate] hydrogels." Tissue Eng Part A **17**(9-10): 1287-1302.

Ropper, A. E., D. K. Thakor, I. Han, D. Yu, X. Zeng, J. E. Anderson, Z. Aljuboori, S.-W. Kim, H. Wang, R. L. Sidman, R. D. Zafonte and Y. D. Teng (2017). "Defining recovery neurobiology of injured spinal cord by synthetic matrix-assisted hMSC implantation." Proceedings of the National Academy of Sciences of the United States of America **114**(5): E820-E829.

Ruzicka, J., N. Romanyuk, A. Hejcl, M. Vetrik, M. Hruby, G. Cocks, J. Cihlar, M. Pradny, J. Price, E. Sykova and P. Jendelova (2013). "Treating spinal cord injury in rats with a combination of human fetal neural stem cells and hydrogels modified with serotonin." Acta Neurobiol Exp (Wars) **73**(1): 102-115.

Sakiyama-Elbert, S., P. J. Johnson, S. I. Hodgetts, G. W. Plant and A. R. Harvey (2012). "Scaffolds to promote spinal cord regeneration." Handbook of clinical neurology **109**: 575-594.

Shanbhag, M. S., J. D. Lathia, M. R. Mughal, N. L. Francis, N. Pashos, M. P. Mattson and M. A. Wheatley (2010). "Neural progenitor cells grown on hydrogel surfaces respond to the product of the transgene of encapsulated genetically engineered fibroblasts." Biomacromolecules **11**(11): 2936-2943.

Sharp, K. G., K. M. Yee and O. Steward (2014). "A re-assessment of long distance growth and connectivity of neural stem cells after severe spinal cord injury." Exp Neurol **257**: 186-204.

Shen, Y., Y. Qian, H. Zhang, B. Zuo, Z. Lu, Z. Fan, P. Zhang, F. Zhang and C. Zhou (2010). "Guidance of olfactory ensheathing cell growth and migration on electrospun silk fibroin scaffolds." Cell Transplant **19**(2): 147-157.

Shi, Q., W. Gao, X. Han, X. Zhu, J. Sun, F. Xie, X. Hou, H. Yang, J. Dai and L. Chen (2014). "Collagen scaffolds modified with collagen-binding bFGF promotes the neural regeneration in a rat hemisected spinal cord injury model." Sci China Life Sci **57**(2): 232-240.

Silva, N. A., J. Moreira, S. Ribeiro-Samy, E. D. Gomes, R. Y. Tam, M. S. Shoichet, R. L. Reis, N. Sousa and A. J. Salgado (2013). "Modulation of bone marrow mesenchymal stem cell secretome by ECM-like hydrogels." Biochimie **95**(12): 2314-2319.

Silva, N. A., N. Sousa, R. L. Reis and A. J. Salgado (2014). "From basics to clinical: A comprehensive review on spinal cord injury." Progress in Neurobiology **114**: 25-57.

Stokols, S. and M. H. Tuszynski (2004). "The fabrication and characterization of linearly oriented nerve guidance scaffolds for spinal cord injury." Biomaterials **25**(27): 5839-5846.

Sun, G. D., J. L. Shao, D. J. Deng, Z. G. Zhou, X. B. Zhou, Y. X. Lin and Z. Z. Li (2017). "A chitosan scaffold infused with neurotrophin-3 and human umbilical cord mesenchymal stem cells suppresses inflammation and promotes functional recovery after spinal cord injury in mice." International Journal of Clinical and Experimental Medicine **10**(8): 11672-11679.

Tang, Z. P., N. Liu, Z. W. Li, X. W. Xie, Y. Chen, Y. H. Shi, W. G. Zeng, S. X. Wang, J. Chen, J. Yang and D. J. Pan (2010). "In vitro evaluation of the compatibility of a novel collagen-heparan sulfate biological scaffold with olfactory ensheathing cells." Chin Med J (Engl) **123**(10): 1299-1304.

Tavakol, S., H. Aligholi, A. Gorji, A. Eshaghabadi, E. Hoveizi, B. Tavakol, S. M. Rezayat and J. Ai (2014). "Thermogel nanofiber induces human endometrial-derived stromal cells to neural differentiation: In vitro and in vivo studies in rat." J Biomed Mater Res A **102**(12): 4590-4597.

Taylor, S. J., J. W. McDonald, 3rd and S. E. Sakiyama-Elbert (2004). "Controlled release of neurotrophin-3 from fibrin gels for spinal cord injury." J Control Release **98**(2): 281-294.

Taylor, S. J., E. S. Rosenzweig, J. W. McDonald, 3rd and S. E. Sakiyama-Elbert (2006). "Delivery of neurotrophin-3 from fibrin enhances neuronal fiber sprouting after spinal cord injury." J Control Release **113**(3): 226-235.

Teng, Y. D., E. B. Lavik, X. Qu, K. I. Park, J. Ourednik, D. Zurakowski, R. Langer and E. Y. Snyder (2002). "Functional recovery following traumatic spinal cord injury mediated by a unique polymer scaffold seeded with neural stem cells." Proc Natl Acad Sci U S A **99**(5): 3024-3029.

Terraf, P., S. M. Kouhsari, J. Ai and H. Babaloo (2017). "Tissue-Engineered Regeneration of Hemisected Spinal Cord Using Human Endometrial Stem Cells, Poly epsilon-Caprolactone Scaffolds, and Crocin as a Neuroprotective Agent." Mol Neurobiol **54**(7): 5657-5667.

Tsai, E. C., P. D. Dalton, M. S. Shoichet and C. H. Tator (2006). "Matrix inclusion within synthetic hydrogel guidance channels improves specific supraspinal and local axonal regeneration after complete spinal cord transection." Biomaterials **27**(3): 519-533.

Tsai, E. C., A. V. Krassioukov and C. H. Tator (2005). "Corticospinal regeneration into lumbar grey matter correlates with locomotor recovery after complete spinal cord transection and repair with peripheral nerve grafts, fibroblast growth factor 1, fibrin glue, and spinal fusion." J Neuropathol Exp Neurol **64**(3): 230-244.

Tukmachev, D., S. Forostyak, Z. Koci, K. Zaviskova, I. Vackova, K. Vyborny, I. Sandvig, A. Sandvig, C. J. Medberry, S. F. Badylak, E. Sykova and S. Kubinova (2016). "Injectable Extracellular Matrix Hydrogels as Scaffolds for Spinal Cord Injury Repair." Tissue Eng Part A **22**(3-4): 306-317.

Tuszynski, M. H. (2002). "Growth-factor gene therapy for neurodegenerative disorders." Lancet Neurology **1**(1): 51-57.

Vesterinen, H. M., E. S. Sena, K. J. Egan, T. C. Hirst, L. Churolov, G. L. Currie, A. Antonic, D. W. Howells and M. R. Macleod (2014). "Meta-analysis of data from animal studies: A practical guide." Journal of Neuroscience Methods **221**: 92-102.

Wang, B., J. Han, Y. Gao, Z. Xiao, B. Chen, X. Wang, W. Zhao and J. Dai (2007). "The differentiation of rat adipose-derived stem cells into OEC-like cells on collagen scaffolds by co-culturing with OECs." Neurosci Lett **421**(3): 191-196.

Wang, H., C. Liu and X. Ma (2012). "Alginic acid sodium hydrogel co-transplantation with Schwann cells for rat spinal cord repair." Arch Med Sci **8**(3): 563-568.

Wang, N., Z. Xiao, Y. Zhao, B. Wang, X. Li, J. Li and J. Dai (2017). "Collagen scaffold combined with human umbilical cord-derived mesenchymal stem cells promote functional recovery after scar resection in rats with chronic spinal cord injury." J Tissue Eng Regen Med.

Wang, N., S. Zhang, A. F. Zhang, Z. Y. Yang and X. G. Li (2014). "Sodium hyaluronate-CNTF gelatinous particles promote axonal growth, neurogenesis and functional recovery after spinal cord injury." Spinal Cord **52**(7): 517-523.

Wang, X., Y. Li, Y. Gao, X. Chen, J. Yao, W. Lin, Y. Chen, J. Liu, Y. Yang and X. Wang (2013). "Combined use of spinal cord-mimicking partition type scaffold architecture and neurotrophin-3 for surgical repair of completely transected spinal cord in rats." J Biomater Sci Polym Ed **24**(8): 927-939.

Wang, Y., C. Zhou, M. Yao, Y. Li, Y. Liu and W. Zheng (2011). "Biodegradable parallel and porous HSPG/collagen scaffolds for the in vitro culture of NSCs for the spinal cord tissue engineering." Journal of Porous Materials **19**(2): 173-180.

Wei, Y. T., Y. He, C. L. Xu, Y. Wang, B. F. Liu, X. M. Wang, X. D. Sun, F. Z. Cui and Q. Y. Xu (2010). "Hyaluronic acid hydrogel modified with nogo-66 receptor antibody and poly-L-lysine to promote axon regrowth after spinal cord injury." J Biomed Mater Res B Appl Biomater **95**(1): 110-117.

Wen, Y., S. Yu, Y. Wu, R. Ju, H. Wang, Y. Liu, Y. Wang and Q. Xu (2016). "Spinal cord injury repair by implantation of structured hyaluronic acid scaffold with PLGA microspheres in the rat." Cell Tissue Res **364**(1): 17-28.

Wen, Y. J., S. K. Yu, Y. H. Wu, R. K. Ju, H. Wang, Y. J. Liu, Y. Wang and Q. Y. Xu (2016). "Spinal cord injury repair by implantation of structured hyaluronic acid scaffold with PLGA microspheres in the rat." Cell and Tissue Research **364**(1): 17-28.

Wilems, T. S., J. Pardieck, N. Iyer and S. E. Sakiyama-Elbert (2015). "Combination therapy of stem cell derived neural progenitors and drug delivery of anti-inhibitory molecules for spinal cord injury." Acta Biomater **28**: 23-32.

Wu, H. F., J. S. Cen, Q. Zhong, L. Chen, J. Wang, D. Y. Deng and Y. Wan (2013). "The promotion of functional recovery and nerve regeneration after spinal cord injury by lentiviral vectors encoding Lingo-1 shRNA delivered by Pluronic F-127." Biomaterials **34**(6): 1686-1700.

Wu, W., S. Y. Lee, X. Wu, J. Y. Tyler, H. Wang, Z. Ouyang, K. Park, X. M. Xu and J. X. Cheng (2014). "Neuroprotective ferulic acid (FA)-glycol chitosan (GC) nanoparticles for functional restoration of traumatically injured spinal cord." Biomaterials **35**(7): 2355-2364.

Xiong, Y., Y. S. Zeng, C. G. Zeng, B. L. Du, L. M. He, D. P. Quan, W. Zhang, J. M. Wang, J. L. Wu, Y. Li and J. Li (2009). "Synaptic transmission of neural stem cells seeded in 3-dimensional PLGA scaffolds." Biomaterials **30**(22): 3711-3722.

Xue, F., E. J. Wu, P. X. Zhang, A. Li-Ya, Y. H. Kou, X. F. Yin and N. Han (2015). "Biodegradable chitin conduit tubulation combined with bone marrow mesenchymal stem cell transplantation for treatment of spinal cord injury by reducing glial scar and cavity formation." Neural Regen Res **10**(1): 104-111.

Yang, E. Z., G. W. Zhang, J. G. Xu, S. Chen, H. Wang, L. L. Cao, B. Liang and X. F. Lian (2017). "Multichannel polymer scaffold seeded with activated Schwann cells and bone mesenchymal stem cells improves axonal regeneration and functional recovery after rat spinal cord injury." Acta Pharmacol Sin **38**(5): 623-637.

Yang, Y., L. De Laporte, C. B. Rives, J. H. Jang, W. C. Lin, K. R. Shull and L. D. Shea (2005). "Neurotrophin releasing single and multiple lumen nerve conduits." J Control Release **104**(3): 433-446.

Yang, Y. H., Z. Khan, C. Ma, H. J. Lim and L. A. Smith Callahan (2015). "Optimization of adhesive conditions for neural differentiation of murine embryonic stem cells using hydrogels functionalized with continuous Ile-Lys-Val-Ala-Val concentration gradients." Acta Biomater **21**: 55-62.

Yang, Z., A. Zhang, H. Duan, S. Zhang, P. Hao, K. Ye, Y. E. Sun and X. Li (2015). "NT3-chitosan elicits robust endogenous neurogenesis to enable functional recovery after spinal cord injury." Proc Natl Acad Sci U S A **112**(43): 13354-13359.

Zaminy, A., M. A. Shokrgozar, Y. Sadeghi, M. Noroozian, M. H. Heidari and A. Piryaei (2013). "Mesenchymal stem cells as an alternative for Schwann cells in rat spinal cord injury." Iran Biomed J **17**(3): 113-122.

Zaminy, A., M. A. Shokrgozar, Y. Sadeghi, M. Norouzian, M. H. Heidari and A. Piryaei (2013). "Transplantation of schwann cells differentiated from adipose stem cells improves functional recovery in rat spinal cord injury." Arch Iran Med **16**(9): 533-541.

Zhang, J., X. Lu, G. Feng, Z. Gu, Y. Sun, G. Bao, G. Xu, Y. Lu, J. Chen, L. Xu, X. Feng and Z. Cui (2016). "Chitosan scaffolds induce human dental pulp stem cells to neural differentiation: potential roles for spinal cord injury therapy." Cell Tissue Res **366**(1): 129-142.

Zhang, L., H. T. Zhang, S. Q. Hong, X. Ma, X. D. Jiang and R. X. Xu (2009). "Cografted Wharton's jelly cells-derived neurospheres and BDNF promote functional recovery after rat spinal cord transection." Neurochem Res **34**(11): 2030-2039.

Zhao, Y. Z., X. Jiang, Q. Lin, H. L. Xu, Y. D. Huang, C. T. Lu and J. Cai (2017). "Thermosensitive heparin-poloxamer hydrogels enhance the effects of GDNF on neuronal circuit remodeling and neuroprotection after spinal cord injury." J Biomed Mater Res A **105**(10): 2816-2829.

Zhao, Y. Z., X. Jiang, J. Xiao, Q. Lin, W. Z. Yu, F. R. Tian, K. L. Mao, W. Yang, H. L. Wong and C. T. Lu (2016). "Using NGF heparin-poloxamer thermosensitive hydrogels to enhance the nerve regeneration for spinal cord injury." Acta Biomater **29**: 71-80.
